# Copper(II)-5-chloro-2-hydroxybenzophenone Complexes with *N*–*N* Donors: Structural Insights and Antitumor Activity against A2780 Human Ovarian Cancer Cells of a Bathophen Derivative

**DOI:** 10.1021/acsomega.5c11889

**Published:** 2026-03-03

**Authors:** Alexandre B. de Carvalho, Marcos V. Palmeira-Mello, Paulo N. de Souza, Saulo H. Mendes Abe, José Balena G. Filho, Marcelo B. Andrade, Rodrigo S. Corrêa, Alzir A. Batista, Javier Ellena

**Affiliations:** † Sao Carlos Institute of Physics, 28133 University of Sao Paulo, IFSC-USP, Sao Carlos, São Paulo 13566-590, Brazil; ‡ Department of Chemistry, Federal University of São Carlos (UFSCar), São Carlos, São Paulo 13565-905, Brazil; § Nanotechnology National Laboratory for Agriculture (LNNA), EMBRAPA Instrumentation, São Carlos, São Paulo 13561-206, Brazil; ∥ Department of Chemistry, Institute of Exact and Biological Sciences, Federal University of Ouro Preto (UFOP), Ouro Preto, Minas Gerais 35402-136, Brazil

## Abstract

Five novel heteroleptic copper­(II) complexes were synthesized and fully characterized. Unlike previously reported Cu­(II)-diimine systems, these complexes incorporate an O,O-chelating 5-chloro-2-hydroxybenzophenone (5-Cl2HBz) ligand, forming cytotoxic compounds active against A2780 (ovarian), A549 (lung), and MCF-7 (breast) cancer cells. The complexes were identified as [Cu­(5-Cl2HBz)­(phen)­(NO_3_)] (Cu­(**1**)), [Cu­(5-Cl2HBz)­(bathophen)]­(NO_3_)­1.5H_2_O·CH_3_OH (Cu­(**2**)), [Cu­(5-Cl2HBz)­(bipy)­(NO_3_)­(H_2_O)] (Cu­(**3**)), [Cu­(5-Cl2HBz)­(5,5′-bipy)­(NO_3_)] (Cu­(**4**)), and [Cu­(5-Cl2HBz)­(*tert*-bipy)­(NO_3_)]·2H_2_O (Cu­(**5**)), where phen = 1,10-phenanthroline, bathophen = 4,7-diphenyl-1,10-phenanthroline, bipy = 2,2′-bipyridine, 5,5′-bipy = 5,5′-bipyridine, and *tert*-bipy = 4,4′-bis­(*tert*-butyl)-2,2′-bipyridine. Among the series, complex Cu­(**2**) displayed outstanding potency in A2780 cells (IC_50_ = 0.24 μM), being 36-fold more potent than cisplatin. This complex significantly affected the cell morphology and colony formation in a concentration-dependent manner. DNA interaction studies revealed that Cu­(**2**) interacts strongly with DNA, as evidenced by viscosity, circular dichroism, and fluorescence measurements. These findings establish Cu­(II)-bathophen derivatives as highly promising candidates for the development of copper-based anticancer agents.

## Graphical abstract

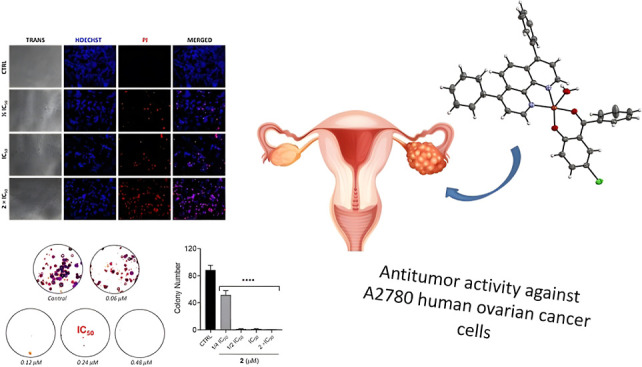

## 1 Introduction

Cancer is a major clinical condition, with the incidence increasing every year due to a variety of factors, including genetic changes, chronic inflammation, unhealthy diets, and even substance abuse. Consequently, cancer cases have been rising rapidly, underscoring the continuous need for novel therapeutic strategies.^1–3^ Through the advancement of cancer research over the years, significant progress has been made in understanding its mechanisms. However, the development of pioneering metal-based drugs with antitumor properties remains a contemporary challenge in the field of metallodrug design.^4^

Platinum complexes are the most commonly used chemotherapeutic agents studied worldwide. These complexes bind covalently to nucleobases of the DNA, triggering apoptotic cell death.^5^ Despite their efficiency, these compounds are associated with several side effects and drug resistance.^6,7^ To overcome these challenges, several metal-based compounds have been investigated for anticancer therapeutic purposes.

In this context, copper­(II) complexes are well-known for their broad range of biological properties and their noteworthy antineoplastic activity.^8,9^ In general, their anticancer activity is often attributed to the synergistic interaction between copper­(II) ions and ligands that already have antitumor activity.^10–13^ The cytotoxic effect of copper­(II) compounds is primarily attributed to the ability of this ion to generate reactive oxygen species (ROS), thereby disrupting key cellular mechanisms related to the redox process. In normal cells, ROS are predominantly produced during mitochondrial respiration. However, in most cellular environments associated with tumor development, ROS levels are markedly elevated. Excessive ROS levels lead to impaired mitochondrial respiration and damage to proteins, nucleic acids, and other cellular components, ultimately resulting in apoptosis.^14–17^

Building on these findings, recent research has focused on the design of heteroleptic copper­(II) complexes combining diimine ligands, such as 1,10-phenanthroline or 2,2′-bipyridine with auxiliary bidentate ligands bearing O–O or O–N donor atoms. This structural motif has proven effective in promoting DNA intercalation, ROS-mediated cytotoxicity, and selective apoptosis in tumor cells, as exemplified by the Casiopein family compounds.^18^ With this framework, hydroxylated benzophenone derivatives stand out as attractive ancillary ligands due to their ability to stabilize metal centers via chelation through phenolic and carbonyl groups, while also introducing photophysical properties and enhanced lipophilicity.^19^ In particular, benzophenones have been explored to modulate the electronic environment and biological reactivity of metal complexes. The incorporation of electron-withdrawing groups, such as chlorine, in the aromatic scaffold can influence the ligand’s donor ability and fine-tune metal–ligand interactions. Notably, copper­(II) complexes incorporating such ligands exhibit remarkable antitumor activity against HepG2 and HCT116 tumor cells.^20^ Furthermore, Levin et al. described that 9-dimethyl-1,10-phenanthroline and 1,10′-phenanthroline Cu­(II)-based compounds exhibit better activity than the latter and cisplatin, against MG-63, A549, MCF-7, and MBA-MB-231 cells.^21^

DNA remains the main target for metal-based compounds. To obtain new insights about the cytotoxicity and DNA-interacting properties of these species, Gamez et al. prepared and studied different copper complexes.^22,23^ Their binding modes were explored using several techniques. The authors revealed that the complex [CuCl_2_(Cltpy)] (Cltpy is 4′-chloro-2,2′:6′,2″-terpyridine) is as efficient a DNA cleaver at 20 μM concentration. Despite the lack of activity after 24 h, this complex has increased cytotoxicity ability after 72 h incubation (120-fold more potent) toward A2780 ovarian cancer cells.^22^ Recently, the authors studied complexes with the general formula [Cu­(en2ampy)]^2+^ (en2ampy is, namely, 3,3′-((1*E*,1′*E*)-(ethane-1,2-diylbis­(azaneylylidene))­bis­(methaneylylidene))­bis­(pyridin-2-amine). Despite their planar structure and IC_50_ values lower than cisplatin, these complexes were not able to intercalate between DNA base pairs, confirming that, as expected, geometric features are not the only parameters involved in these interactions.^23^

Herein, we explore the cytotoxicity of copper­(II) complexes with a 5-chloro-2-hydroxybenzophenone and N–N donors. Accordingly, we report the synthesis of five Cu­(II)-5-chlorobenzophenone-based compounds containing different N-heterocyclic ligands. These compounds, namely, [Cu­(5-Cl2HBz)­(phen)­(NO_3_)]—Cu­(**1**), [Cu­(5-Cl2HBz)­(bathophen)]·(NO_3_)·1.5 H_2_O·CH_3_OH—Cu­(**2**), [Cu­(5-Cl2HBz)­(bipy)­(NO_3_)­(H_2_O)]—Cu­(**3**), [Cu­(5-Cl2HBz)­(5,5′-bipy)­(NO_3_)]—Cu (**4**), and [Cu­(5-Cl2HBz)­(*tert*-bipy)­(NO_3_)]·2H_2_O—Cu (**5**), where (5-Cl2HBz = 5-chloro-2-hydroxybenzophenonephenone, phen = 1,10-phenanthroline, bathophen = 4,7-diphenyl-1,10-phenanthroline, bipy = 2,2′-bipyridine, 5,5′-bipy = 5,5′-bipyridine and *tert*-bipy = 4,4′-bis­(*tert*-butyl)-2,2′-bipyridine). These complexes were fully characterized by elemental analysis, thermogravimetry, infrared spectroscopy, single-crystal X-ray diffraction, UV–vis spectroscopy, ESI-MS, and EPR.

Their cytotoxic potential was explored in different cancer cell lines. Due to the promising results, complex Cu­(**2**) was further studied in A2780 human ovarian cancer cells. A clonogenic assay was also performed. Furthermore, metal complex–DNA interactions were studied. Complex Cu­(**2**) was found to interact with the biomolecule via different modes, making it a promising starting point for further development of new cytotoxic copper-bathophen agents.

## 2 Experimental Section

### 2.1 Materials

Solvents and all chemicals used were of reagent grade or comparable purity and were supplied and used as received from Sigma-Aldrich: copper­(II) nitrate trihydrate, 1,10-phenanthroline (phen), 4,7-diphenyl-1,10-phenanthroline (bathophen), 2,2′-bipyridine (bipy), 5,5′-bipyridine (5,5′-bipy), 4,4′-bis­(*tert*-butyl)-2,2′-bipyridine (*tert*-bipy), 5-chloro-2-hydroxybenzophenone (5-Cl2HBz), and calf-thymus DNA (CT-DNA).

### 2.2 Instrumentation

Partial elemental analyses were performed on a CHNS Thermo Scientific Fisons Instruments EA 1108 model CHNS analyzer. Conductivity data (present as Ω^–1^ cm^2^ mol^–1^) were obtained in methanol by using a Bel Engineering conductivity meter with a cell constant equal to 1.25 cm^–1^. Measurements were made at room temperature (18 °C) using a 10 mM solution. Thermogravimetric analyses were carried out using Shimadzu thermal analysis equipment, model 60H, with an alumina sample holder, in a N_2_ atmosphere, with a flow rate of 50 mL min^–1^ and a heating rate of 10 °C min^–1^. A Bomem-Michelson FT-IR spectrophotometer was employed to record infrared spectra (4000 cm^–1^–400 cm^–1^) using KBr plates. The Raman spectra data collection was carried out using a HORIBA LabRAM HR Evolution spectrometer equipped with a 532 nm laser, a grating of 1800 gr/mm, and a CCD cooled detector. Laser power at the sample was 1 mW. The spectra were collected with a resolution of 1 cm^–1^. The electronic absorption spectra were acquired in methanol and DMSO/buffer pH = 7.4 solution using a Shimadzu 1800 UV–vis spectrophotometer with cuvettes with an optical path of 1 cm containing solutions of the complexes and ligands of known concentrations. Full mass spectra in positive ion mode were acquired on a MicroTOF-Q Bruker equipped with electrospray mass ionization and an ion trap analyzer. Samples were dissolved in MeOH in a 10 mM solution and continuously pumped by a syringe (Hamilton 500 μL) with a flow of 15 μL min^–1^ into the mass spectrometer. The positive ion mode electrospray was achieved by application of +4.5 kV and 180 °C desolvation temperature. The EPR measurements were performed in a X-band spectrometer from Bruker, frequency 9.8 GHz. Each complex spectrum was recorded at room temperature by extracting 50 μL of a 10 mM solution through a glass capillary tube and placing it in a regular X-band EPR quartz tube. The parameters used were: 100 kHz of modulation frequency, 1 G of modulation amplitude, and 3.65 mW of microwave power. Two software packages were used to analyze spectrometry data (a) mmass (open-source mass spectrometry tool) and (b) Envipat.^24^

### 2.3 General Procedure to Synthesize the Heteroleptic Copper­(II) Complexes

The general synthetic route used to synthesize the heteroleptic copper­(II) complexes with the general formula [Cu­(5-Cl2HBz)­(N–N)­(NO_3_)], where N–N = phen—Cu­(**1**), bathophen—Cu­(**2**), bipy—Cu­(**3**), 5,5′-bipy—Cu­(**4**), and *tert*-bipy—Cu­(**5**), is described in Scheme 1. In a round-bottomed flask (50 mL), the Cu­(NO_3_)_2_ · 2.5 H_2_O (58 mg, 0.25 mmol) was dissolved in methanol (10 mL). A methanolic solution of 5-Cl2HBz (58 mg, 0.25 mmol) was deprotonated by using KOH (20 mg, 0.35 mmol). The mixture was stirred for 20 min and, afterward, a methanolic solution containing an N-heterocycle (0.25 mmol) was added dropwise, and the resulting solution was kept under reflux with stirring for 24 h. The crystals were obtained by slow evaporation after 2 weeks. The obtained green crystals were washed with ultrapure cold water to remove impurities and dried under pressure.

**Scheme 1:**
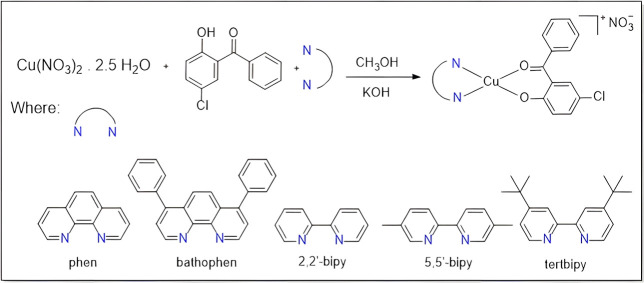
General Synthetic Route Used to Obtain the Copper­(II) Complexes Cu­(**1**)–Cu­(**5**)

[Cu­(5-Cl2HBz)­(phen)­(NO_3_)]—Cu­(**1**). Yield: 78%. Green crystal (FW = 537.4 g mol^–1^). Elemental analysis for (CuC_25_H_16_ClN_3_O_5_) Calcd: 55.87%; H, 3.00%; N, 7.82%. Found: C, 55.49%; H, 2.81%; N, 7.78%. Molar conductivity: (1 × 10^–3^ molL^–1^, MeOH): 108 Ω^–1^ cm^2^ mol^–1^. ESI-MS (CuC_25_H_16_N_3_O_5_Cl [M]^+^) calcd, 474.0196; found, 474.0339. IR (KBr, cm^–1^): 3060, 2990, 1645, 1592, 1505, 1417, 1338, 1143, 1090, 857, 737. UV–vis (methanol), λ_max_/nm (ε/M^–1^ cm^–1^): 272 (17,000), 294 (16,000), 419 (146).

[Cu­(5-Cl2HBz)­(bathophen)]·(NO_3_)·1.5 H_2_O·CH_3_OH—Cu­(**2**). Yield: 68%. Dark green crystal (FW = 707.62 g mol^–1^). Elemental analysis for (C_76_H_62_Cl_2_Cu_2_N_6_O_15_): C, 58.84%; H, 4.42%; N, 5.42%. Found: C, 59.29%; H, 4.12%; N, 5.42%. Molar conductivity: (1 × 10^–3^ molL^–1^, MeOH): 97.5 Ω^–1^ cm^2^ mol^–1^. ESI-MS (CuH_24_ClN_2_O_2_ [M^+^]) calcd, 628.0973; found, 628.1232. IR (KBr, cm^–1^): 3050, 3023, 1606, 1553, 1509, 1487, 1404, 1078, 924, 845, 765, 704. UV–vis (methanol), λ_max_/nm (ε/M^–1^ cm^–1^): 220 (15,600), 285 (1470), 420 (102).

[Cu­(5-Cl2HBz)­(bipy)­(NO_3_)­(H_2_O)]—Cu­(**3**). Yield: 82%. Green crystal (FW = 531.40 g mol^–1^). Elemental analysis for (CuC_23_H_18_ClN_3_O_6_): C, 51.98%; H, 3.41%; N, 7.91%. Found: C, 52.35%; H, 3.19%; N, 7.94. Molar conductivity: (1 × 10^–3^ molL^–1^, MeOH): 57.2 Ω^–1^ cm^2^ mol^–1^. ESI-MS: (CuC_23_H_16_ClCuN_2_O_2_ [M^+^]) calcd, 452.0347; found, 452.0311. IR (KBr, cm^–1^): 3088, 3055, 1577, 1549, 1451, 1413, 1258, 1080, 982, 762, UV–vis (methanol) λ_max_/nm (ε/M^–1^ cm^–1^): 238 (15,600), 297 (1010).

[Cu­(5-Cl2HBz)­(5,5′-bipy)­(NO_3_)]—Cu­(**4**). Yield: 77%. Green crystal (FW = 541.43 g mol^–1^). Elemental analysis for (CuC_25_H_20_N_3_O_5_Cl): C, 55.46%; H, 3.72%; N, 7.33%. Found: C, 56.31%; H, 3.36%; N, 7.13. Molar conductivity: (1 × 10^–3^ molL^–1^, MeOH): 183.2 Ω^–1^ cm^2^ mol^–1^. ESI-MS: (CuC_25_H_20_ClN_2_O_2_ [M^+^]) calcd, 480.066; found, 480.0884. IR (KBr, cm^–1^): 3005, 2950, 1596, 1548, 1496, 1373, 1271, 1218, 1120, 1024, 820, 732, 660. UV–vis (methanol) λ_max_/nm (ε/M^–1^ cm^–1^): 251 (9300), 308 (8300), 413 (400).

[Cu­(5-Cl2HBz)­(*tert*-bipy)­(NO_3_)]·2H_2_O—Cu­(**5**). Yield: 75%. Green crystal (FW = 661.62 g mol^–1^): Elemental analysis for (C_31_H_36_ClCuN_3_O_7_): C, 56.27%; H, 5.48%; N, 6.35%. Found: C, 57.23%; H, 4.49%; N, 6.07%. Molar conductivity: (1 × 10^–3^ mol L^–1^, MeOH): 42.2 Ω^–1^ cm^2^ mol^–1^. ESI: (CuC_31_H_32_ClN_2_O_2_ [M^+^]) calcd, 562.1448; found, 564.1911 *m*/*z*. IR (KBr, cm^–1^): 3066, 2972, 1623, 1604, 1468, 1379, 1280, 1158, 951, 824. UV–vis (methanol): λ_max_/nm (ε/M^–1^ cm^–1^): 284 (2699), 296 (2682), 307 (2269), 415 (488).

### 2.4 Single-Crystal X-ray Diffraction

X-ray diffraction data were collected on a Rigaku XtaLAB Synergy-S Dualflex diffractometer equipped with a HyPix 6000HE detector, using Cu Kα radiation (1.54184 Å). The crystal was kept at a steady *T* = 100.0(2) K during data collection using an Oxford Cryosystems 800 Series Cryostream Cooler. CrysAlisPro was used for data collection and reduction, cell refinement, and absorption correction.^26^ The solution of the structures was performed using the Intrinsic Phasing method from SHELXT-2018/2 program,^27^ while the refinement was conducted using the full matrix least-squares on *F* ^2^ using the SHELXL-2019/2 program^28^ with both hosted on Olex2 system.^29^ Non-hydrogen atoms were refined by considering anisotropic displacement parameters, while the hydrogen atoms were refined isotropically at idealized positions using the riding model. Structures were deposited in the Cambridge Structural Database^30^ under CCDC numbers 2475351, 2475352, 2475353, 2475354, and 2475355, for complexes Cu­(**1**), Cu­(**2**), Cu­(**3**), Cu­(**4**), and Cu­(**5**), respectively.

### 2.5 Cell Culture

The complexes were tested against A2780 human ovarian carcinoma cells (ECACC 93112519), A549 human lung cancer (ATCC CCL-185), MCF-7 human breast cancer cells (ATCC HTB-22), and noncancer lung cells MRC5 (ATCC CCL-171). The cells were routinely maintained with Dulbecco’s modified Eagle medium (DMEM; A549 and MRC-5) or Roswell Park Memorial Institute 1640 medium (RPMI 1640; A2780 and MCF-7) supplemented with 10% fetal bovine serum (FBS), at 37 °C in a humidified 5% CO_2_ atmosphere. Cells were obtained from the Rio de Janeiro Cell Bank (BCRJ). Cell culture media and FBS were obtained from Vitrocell and Gibco, respectively.

### 2.6 In Vitro Cytotoxicity Assay

The cytotoxic of complexes Cu­(**1**)–Cu­(**5**) was investigated via 3-(4,5-dimethylthiazol-2-yl)-2,5-diphenyltetrazolium bromide (MTT) assay.^31^ Cells were seeded in 150 μL of an appropriate medium in 96-well plates and then incubated at 37 °C in 5% CO_2_ for 24 h. The compounds were dissolved in DMSO, and 0.75 μL was added to wells (final concentration of 0.5% DMSO/well). Cells were incubated with the compounds for 48 h at 37 °C in 5% CO_2_. Then, 50 μL of MTT (1 mg mL^–1^ in PBS pH 7.4) was added to each well. Cells were incubated again for 4 h, the medium was removed, and formazan crystals were solubilized in DMSO (150 μL). The absorbance was measured by using a BioTek Epoch microplate spectrophotometer at 540 nm. All compounds were tested in three independent experiments performed in triplicate. DMSO was used as the negative control. The cell viability (IC_50_) was determined using GraphPad Prism 8.0.2 software.

### 2.7 Clonogenic Assay

Cells (0.8 × 10^3^ cells per well) were seeded in a 6-well plate and then incubated at 37 °C in 5% CO_2_ for 24 h. After this period, the cells were treated with complex Cu­(**2**) at 1 × IC_50_, 1/2 × IC_50_, IC_50_, and 2 × IC_50_ concentrations (0.06–0.48 μM) and incubated for an additional 48 h. After this period, RPMI medium was replaced with fresh medium, and the plates were incubated for an additional 10 days. Then, the culture medium was removed, and the colonies formed were washed with PBS, fixed with a methanol/acetic acid (3:1) solution, and stained with violet crystal 0.5% in methanol for 30 min. Further, the plates were washed with water and dried. The images were taken using an Invitrogen iBright 1500 Imaging System (Thermo Fisher). The experiment was performed in triplicate. The number of colonies was obtained using ImageJ software, as previously reported.^32^

### 2.8 Cell Morphology and Double Staining Assays

The morphological changes induced on A2780 were studied after treatment with complex Cu­(**2**). Cells (1 × 10^4^ cells per well) were seeded in a 96-well plate and incubated at 37 °C in a humidified atmosphere containing 5% CO_2_ for 24 h. After this period, complex Cu­(**2**) was added at 1/2 × IC_50_, IC_50_, and 2 × IC_50_ concentrations (0.12–0.48 μM). For the fluorescence images, the cells were incubated with Hoechst 33258 and propidium iodide (PI) for 30 min in the dark. All images were obtained using a CELENA S Digital Imaging System (Logos Biosystems).

### 2.9 DNA-Interacting Properties

For the DNA-binding studies, the concentration of calf thymus DNA (CT-DNA, Sigma-Aldrich) was determined spectrophotometrically at 260 nm using the nucleobase molar absorptivity of 6600 L mol^–1^ cm^–1^.

### 2.10 Viscosity Assay

The viscosity experiments were carried out by using an Ostwald viscosimeter maintained in a thermostatic bath at 25 °C. The samples (2.0 mL) were prepared in Tris-HCl buffer (pH 7.4) containing 10% DMSO. The CT-DNA concentration was kept constant at 100 μM, and the concentrations of complexes were varied to obtain different molar ratios, [complex]/[CT-DNA] (0.10–0.75). The final mixture complex/DNA was incubated at 37 °C for 1 h. The flow times were recorded with a digital stopwatch in five replicates. The specific viscosity values (η/η_0_)^1/3^ were plotted versus [complex]/[CT-DNA], where η and η0 correspond to the relative viscosity of DNA in the presence and the absence of the complex, respectively. The equation η_0_ = (*t* – *t*_0_)/*t*_0_ was used to calculate the relative viscosity of DNA (η_0_) values from the flow time of the DNA solution (*t*) corrected for the flow time of the buffer (*t*_0_). Thiazole orange and cisplatin were used as controls.

### 2.11 Circular Dichroism

The CD titrations were carried out using a JASCO J-815 spectropolarimeter at 25 °C. Solutions of CT-DNA (100 μM) in Tris-HCl buffer (pH 7.4) containing 10% DMSO with different molar ratios [complex]/[CT-DNA] (0.25–1.0) were incubated at 37 °C for 24 h. A total of 4 accumulations of the spectra were recorded from 230 to 500 nm using a quartz cuvette with an optical path length of 0.5 cm, and a scanning rate of 200 nm min^–1^. The measurements were performed at the University of São Paulo (USP), São Carlos—SP, Brazil.

### 2.12 Fluorescence Dye Displacement Assay

A solution of CT-DNA (100 μM) was preincubated with EB (100 μM) in Tris-HCl buffer (pH 7.4) for 1 h at 37 °C to allow full interaction of the dye with the biomolecule. Increasing amounts of Cu­(**2**) (10–100 μM) were subsequently added to the DNA samples, followed by incubation for 24 h. Fluorescence spectra were registered from 370 to 700 nm at 25 °C upon excitation at 510 nm by using a Synergy/H1-Biotek fluorimeter. For data analysis, the classical Stern–Volmer equation was used: *F*_0_/*F* = 1 + *K*_sv_ [*Q*], where *F*_0_ and *F* represent the fluorescence intensities of the DNA–dye complex in the absence and presence of a quencher, respectively. *K*_sv_ is the linear Stern–Volmer quenching constant and [*Q*] is the concentration of the added complex.

The experiment was performed in triplicate, and the *K*_sv_ was obtained via linear regression and reported as means ± SD.

### 2.13 Agarose Gel Electrophoresis

Agarose gel electrophoresis using pBR322 plasmid DNA was performed to obtain insights into the cleavage potential of Cu­(**2**). A stock solution of the complex in DMSO was prepared and diluted in the Tris-HCl buffer (pH 7.4). The plasmid pBR322 (100 μM) was treated with different concentrations of Cu­(**2**) (10, 20, 40, 60, 80, and 100 μM), and the samples were incubated at 298 K for 1 h. Samples of free DNA and cisplatin (100 μM) were used as the controls. A gel was prepared using agarose (1%) in TAE buffer 1× (Tris-acetate-EDTA), and the samples were loaded with 10 μL of loading buffer (30% glycerol, 5 mM xylene cyanol). The gel was run in a TAE 1× at 50 V and 40 mA for 2 h in a Bio-Rad horizontal tank. Furthermore, the gel was stained with ethidium bromide (EB) during 1 h, and the image was obtained using a Gel Doc EZ Imager instrument (Bio-Rad).

## 3 Results and Discussion

### 3.1 Synthesis and Characterization

Complexes Cu­(**1**)–Cu­(**5**) were obtained by the reaction of Cu­(NO_3_)_2_ · 2.5 H_2_O with the ligands 1,10-phenanthroline (phen), 4,7-diphenyl-1,10-phenanthroline (bathophen), 2,2′-bipyridine (bipy), 5,5′-bipyridine (5,5′-bipy), 4,4′-bis­(*tert*-butyl)-2,2′-bipyridine (*tert*-bipy), and 5-chloro-2-hydroxybenzophenone (5-Cl2HBz) (see Scheme 1).

The copper­(II) complexes Cu­(**1**)–Cu­(**5**) were obtained as air-stable green crystals with satisfactory yields (70 to 80%). Molar conductivity measurements indicated 1:1 electrolyte behavior for all compounds, with NO_3_^–^ as the counterion.^33^ Furthermore, % of C, H, and N elemental analysis results for complexes Cu­(**1**)–Cu­(**5**) agree with the proposed structures. Suitable crystals of all complexes grew up by slow diffusion of a methanol-dichloromethane solution, and their structures were determined by single-crystal X-ray diffraction analysis. The presence of crystallization water molecules in Cu­(**2**), Cu­(**3**), and Cu­(**5**) were confirmed by the thermogravimetric curves, which show a weight loss of 3.81% (calcd, 3.81%) for Cu­(**2**), 3.11% (calcd, 3.38%) for Cu­(**3**), and 5.21% (calcd, 5.44%) for Cu­(**5**) (Figures S1–S3, Supporting Information).

The FT-IR spectra for Cu­(**1**)–Cu­(**5**) exhibited bands at approximately 3069 cm^–1^, corresponding to aromatic C–H stretching vibrations from the N–N heterocyclic derivatives and 5-Cl2HBz ligands. The C=O stretching vibrations were shifted from the observed 1625 cm^–1^ in the free 5-Cl2HBz ligand to lower wavenumbers at 1606 cm^–1^ for Cu­(**2**) and 1596 cm^–1^ for Cu­(**4**) upon coordination, suggesting binding to the copper­(II) metallic center. The nitrate molecule presents two bands, which may indicate coordination to the metallic center. These bands are observed in regions from 765 to 799 cm^–1^ and from 1370 to 1390 cm^–1^, which are references to the stretching υ­(ONO) and the deformation of structure δ­(ONO), respectively (see Figures S4–S8, Supporting Information). In a copper­(II) nitrate complex where the nitrate ion adopts a monodentate coordination mode, the Cu–O stretching vibration in Raman spectra typically appears in the range of 310–350 cm^–1^.^34^ This vibrational mode is attributed to the bond between the copper­(II) center and an oxygen atom of the nitrate group. In the case of complexes Cu­(**1**)–Cu­(**5**), the Cu–O stretching vibration of nitrate is observed between 300 and 400 cm^–1^ (see Figures S9–S13, Supporting Information).

The UV–vis absorption spectra of complexes Cu­(**1**)–Cu­(**5**) in methanol exhibited a distinct absorption band with a maximum ranging from 222 to 273 nm, corresponding to π–π* transitions associated with intraligand (IL) electronic excitations. Additionally, absorption features observed between 355 and 423 nm were assigned to metal-to-ligand charge transfer (MLCT) (see Figures S14–S18, Supporting Information). The presence of complexes in a solution of methanol was investigated by ESI–MS in positive ion mode. The mass spectra of complexes Cu­(**1**)–Cu­(**5**) (Figure S19–S23, Supporting Information) show the main [M]^+^ experimental (theoretical) monoisotopic signals at *m*/*z* 474.0199 (474.0196); 626.0953 (626.0822); 450.0215 (450.0186); 478.0539 (478.0509); and 562.1414 (562.1448) Da, respectively.

### 3.2 Crystal Structure Determination of Cu­(**1**)–Cu­(**5**)

Single crystals of all complexes were obtained by slow evaporation from a methanolic/dichloromethane solution (1:2 ratio). The complexes Cu­(**1**), Cu­(**2**), Cu­(**4**), and Cu­(**5**) crystallize in the triclinic system (space group P1̅ ), while the complex Cu­(**3**) crystallizes in the orthorhombic space group *Pbca*. The crystallographic parameters of complexes Cu­(**1**)–Cu­(**5**) are summarized in Table 1.

**Table 1:** Crystallographic Data and Structure Refinement Parameters of Complexes Cu­(**1**)–Cu­(**5**)

complex	Cu(**1**)	Cu(**2**)	Cu(**3**)	Cu(**4**)	Cu(**5**)
formula	C_25_H_16_N_3_O_5_ClCu	C_76_H_62_C_12_Cu_2_N_6_O_15_	C_23_H_18_ClCuN_3_O_6_	C_25_H_20_N_3_O_5_ClCu	C_31_H_36_ClCuN_3_O_7_
*D*_calc_/g cm–^3^	1.694	1.500	1.636	1.559	1.405
μ/mm–^1^	3.038	2.165	2.992	2.777	2.197
formula weight	537.40	1497.29	531.39	541.43	661.62
crystal system	triclinic	triclinic	orthorhombic	triclinic	triclinic
space group	P1̅	P1̅	*Pbca*	P1̅	P1̅
*a*/Å	7.22830(10)	10.2143(2)	8.11950(10)	8.5987(2)	8.89920(10)
*b*/Å	12.4907(3)	13.2021(3)	18.64350(10)	10.3416(3)	12.7745(2)
*c*/Å	13.4614(4)	13.3361(3)	28.4988(2)	13.8245(3)	14.4465(2)
α/deg	62.674(3)	105.452(2)	90	110.032(2)	104.4510(10)
β/deg	85.087(2)	98.191(2)	90	92.747(2)	92.4560(10)
γ/deg	77.422(2)	101.979(2)	90	90.590(2)	99.5700(10)
*V*/Å^33^	1053.70(5)	1657.90(7)	4314.03(7)	1153.17(5)	1562.10(4)
*Z*	2	1	8	2	2
*Z*′	1	0.5	1	1	1
θ_min_/deg	6.274	4.526	3.101	4.553	5.060
θ_max_/deg	70.062	70.071	79.329	74.485	70.065
measured refl.	17787	33975	33167	23692	54260
independent refl.	3939	6282	4644	4681	5941
reflections with *I* > 2(*I*)	3735	5732	4376	4287	5585
*R*_int_	0.0327	0.0378	0.0339	0.0377	0.0437
ref. parameters	316	388	308	318	419
GooF	1.076	1.072	1.074	1.041	1.037
w*R*_2_ (all data)	0.0751	0.0917	0.0859	0.1133	0.1043
w*R*_2_	0.0744	0.0898	0.0846	0.1096	0.1025
*R*_1_ (all data)	0.0297	0.0352	0.0324	0.0418	0.0400
*R*_1_	0.0284	0.0323	0.0308	0.0390	0.0381

The crystal structure of complex Cu­(**1**) is shown in Figure 1 and crystallographic structures for Cu­(**2**), Cu­(**3**), Cu­(**4**), and Cu­(**5**) are presented in Supporting Information (Figure S24). The X-ray diffraction studies confirm the bidentate coordination of the diimine and 2-hydroxybenzophenone derivatives in all complexes. The structural data are consistent with results obtained for previously reported compounds.^20^ For complex Cu­(**2**), the solvation sites are occupied by a nitrate ion, 1.5 water molecules, and one methanol molecule.

**Figure 1:**
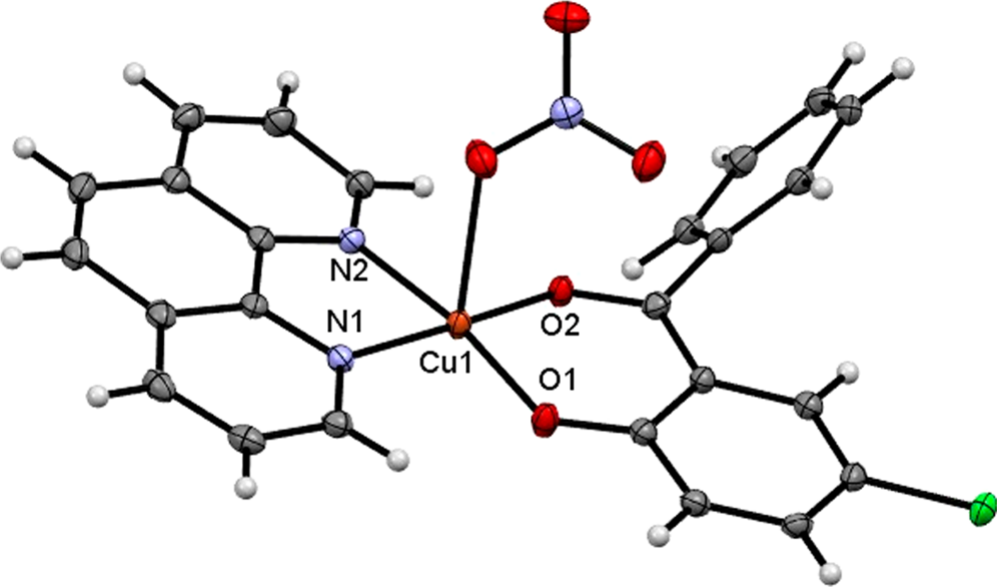
ORTEP-type view and atomic numbering of Cu­(**1**), showing atom labeling and 50% probability ellipsoids.

The coordination geometries of the copper­(II) complexes were analyzed using τ parameter introduced by Addison et al., which provides a quantitative measure for distinguishing between square-pyramidal and trigonal-bipyramidal arrangements in five-coordinated species. The bond distances and angles of Cu­(**1**)–Cu­(**5**) are summarized in Table S1 (Supporting Information). The τ parameter was calculated as τ = (β – α)/60, where β and α represent the largest and second-largest coordination angles, respectively.^35^ As shown in Table 2, the Cu­(**1**) and Cu­(**5**) complexes present τ values of 0.03 and 0.04, indicating nearly ideal square-pyramidal geometries. Complexes Cu­(**3**) and Cu­(**4**) display slightly higher τ values (0.14 and 0.12), consistent with moderately distorted square-pyramidal structures. The complex Cu­(**2**), with a coordination number of four, was not analyzed by this method, but its bond angles are characteristic of a distorted square planar environment, as indicated by two nearly linear *trans* and *cis* axes close to 90°.

**Table 2:** Predominant Geometry for the Cu (**1**–**5**), Angles β, and α and τ Values

complex	coord. number	β (deg)	α (deg)	τ	predominant geometry
Cu (**1**)	5	173.09	171.17	0.03	nearly ideal square-pyramidal
Cu (**2**)	4	--	--	--	distorted square-planar
Cu (**3**)	5	172.22	163.85	0.14	distorted square-pyramidal
Cu (**4**)	5	168.77	161.81	0.12	distorted square-pyramidal
Cu (**5**)	5	171.42	169.03	0.04	nearly ideal square-pyramidal

Notably, the complex Cu­(**3**) exhibits a coordinated water molecule instead of a nitrate ion. The Cu–O1 bond distances range from 1.8808(12) Å to 1.9255(14) Å across the complexes. The C7=O2 bond length in the coordinated ligands (1.250(2) Å to 1.260(19) Å) is slightly elongated compared to the free ligand 1.2392(16) Å, indicating bond lengthening upon coordination. Similarly, the C2–O1 bond distance in the coordination ligands 1.295(19) Å to 1.301(2) Å is shorter than in the free ligand 1.3506(16) Å, suggesting electron delocalization within the structural moiety.^36^ All copper­(II) complexes feature a 5-chloro-2-hydroxybenzophenone ligand forming a six-membered chelate ring on the one side and an N-heterocyclic ligand forming a five-membered ring on the other side. In the axial positions of Cu­(**1**), Cu­(**4**), and Cu­(**5**), a nitrate ion coordinates in a monodentate mode. The Cu–O3 bond distances 2.2352(16) Å to 2.4220(14) Å are significantly longer than the equatorial bonds, consistent with the Jahn–Teller effect. This elongated axial bonding weakens the Cu–O3 interaction, explaining the observed ionic labilization of the nitrate group in solution, which dissociates to form an electrolyte species. Table S1 summarizes the geometrical parameters for Cu­(**1**)–Cu­(**5**).

To better understand the noncovalent interactions within the complex structures, the Hirshfeld surface (HS) analysis, a key method for investigating intermolecular interactions in crystalline solids, was performed. The HS and two-dimensional fingerprint plots for Cu­(**1**), Cu­(**3**), and Cu­(**4**) complexes HS were obtained from the crystallographic information files (CIFs) generated by SCXRD analyses using the CrystalExplorer 17.5 program package.^37^ The HS of Cu­(**2**) and Cu­(**5**) could not be generated due to the application of solvent mask use. The *d*_norm_ surfaces were mapped over the color scale from −0.7 (red) to 1.3 (blue), and the shape index surfaces were obtained in the range of −1.0 (red) to 1.0 (blue) The bidimensional fingerprint plots were generated with the combination of the di and de distances in the scale of 0.4 to 2.8 Å. One of the important characteristics of high-efficient stability of complexes is the presence of π–π interactions in the crystalline structure due to the presence of several rings in the ligands used aligned by the intermolecular interactions, as exemplified by complex Cu­(**1**) in Figure S25 (Supporting Information). In Figure 2, the strongest contacts (C–H···O, C···C, O–H···O, C– H···C, and C–H···N) are highlighted.

**Figure 2:**
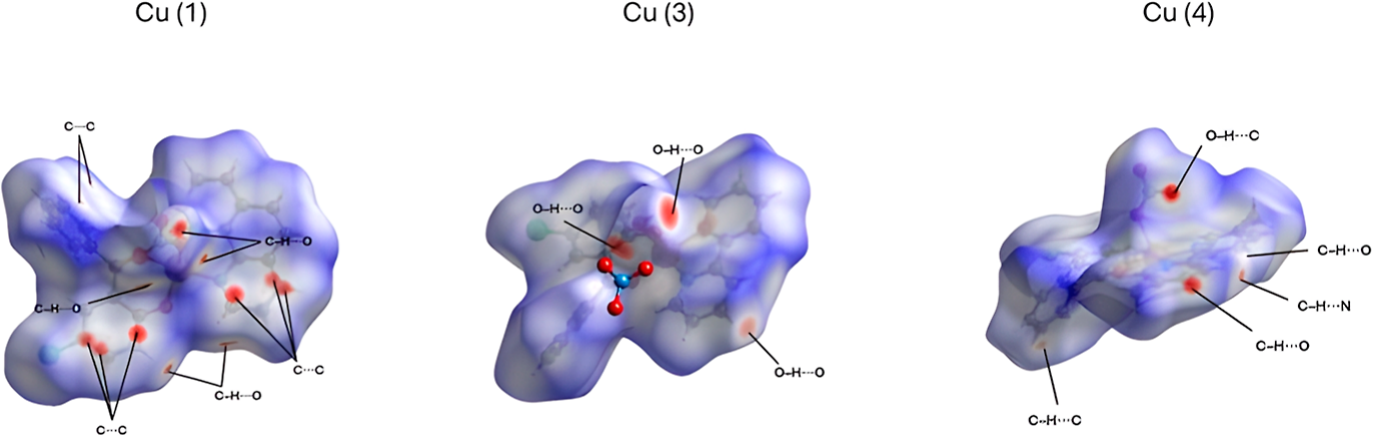
Hirshfeld surface of Cu (**1**), Cu­(**3**), and Cu­(**4**) mapped in *d*_norm_.

The Cu­(**1**) and Cu­(**3**) cation surfaces were obtained by mapping them using the *d*_norm_ function, which is obtained through the combination of the normalized distances from the nearest atom outside (de) and inside (di) the surface. The *d*_norm_ surface shows regions in red, white, and blue to indicate contacts with distances shorter and longer than the sum of the vdW radii of the involved atoms, respectively. For Cu­(**1**) and Cu­(**4**), it is possible to observe the presence of non-classical C–H···O interactions, only in Cu­(**1**) in the structure, as the C···C contacts are related to the π–π interactions between the rings, as verified in the shape index surfaces.

The 2D-fingerprint plots of the complexes (Figure S26–28, Supporting Information) clearly show the intermolecular contacts present in the structures, and its decomposition allows the identification of the contribution for each contact to the crystal packing, showing that the major contributions for the complexes were H···H and O···H contacts. The H···H contact comprises 28.7%, 36.0%, and 32.7% for Cu­(**1**), Cu­(**3**), and Cu­(**4**), respectively. Meanwhile the O···H contact represented 11.2% Cu­(**1**), 14.7% Cu­(**3**), and 11.6% Cu­(**4**). Furthermore, the C···C contact is more prominent in the structures with strong π–π interactions, as in the Cu­(**1**) complex where these contact comprise 11.4%, in contrast with the other two complexes (Cu­(**3**) and Cu (**4**)) where they represent only 5.5% and 4.2%, respectively (Figure S29, Supporting Information).

### 3.3 EPR Spectroscopy for Cu­(**1**)–Cu­(**5**)

Given that Cu­(II) is paramagnetic (S = 1/2), complexes Cu­(**1**)–Cu­(**5**) were investigated by EPR spectroscopy. All compounds exhibit similar effective g values (*g*_eff_ ∼ 2.07), while distinct spectral features are observed in their respective transitions. Complexes Cu­(**1**), Cu­(**2**), Cu­(**3**), and Cu­(**5**) exhibit four well-defined main resonant lines, which can be attributed to the interaction of the unpaired electron with the nuclear spin of copper (^63^Cu and ^65^Cu, *I* = 3/2), due to the *n* = 2*I* + 1 rule.^38^ These signals have different intensities because of the anisotropy arising from the long correlation time (τ), which is mainly associated with the size of the ligands since methanol is not a solvent with sufficient viscosity to restrict the complex motion. In the case of complexes Cu­(**2**), Cu­(**3**), and Cu­(**5**), the transitions related to the interaction of the unpaired electron with the ^14^N nucleus of the nitrogen ligands (*I* = 1) are also evident.^39^ The complex Cu­(**4**) presented the most distinct signal of the entire series, showing a broad spectrum that may be associated with exchange interactions. Since all EPR spectra were recorded at the same molarity and temperature, the authors hypothesize that in solution this species underwent dimerization between its units, favoring a more effective interaction between the Cu­(II) centers, contributing to line broadening (Figures S30–S34, Supporting Information).

### 3.4 Stability in Solution

The stability of the complexes was studied prior to biological investigations. The experiments were conducted by monitoring the spectra of the complexes in aqueous solutions. The spectra of complexes Cu­(**1**)–Cu­(**5**) were recorded in Tris buffer containing 1% of DMSO. No changes were observed for up to 72 h in all cases indicating that the complexes were stable under these conditions (Figures S19–S23). It is important to emphasize that no significant changes were observed in the spectra during stability experiments, reaffirming that they remain stable (Figure S35–S39).

### 3.5 Biological Investigations

The anticancer activity of copper complexes Cu­(**1**)–Cu­(**5**) was investigated in three human cancer cell lines: A2780 (ovarian), A549 (lung), and MCF-7 (breast), as well in the nontumorigenic MRC-5 lung cells following 48 h of incubation time. The cytotoxicity data, expressed as IC_50_ ± SD (μM), are presented in Table 3. Overall, these complexes were cytotoxic in all cancer cells tested. In general, complexes Cu­(**1**)–Cu­(**5**) exhibited increased cytotoxicity in comparison to the Cu­(NO_3_)_2_ starting material and all free ligands. Moreover, complex Cu (**2**) presented a high antitumor effect in all cell lines, particularly in ovarian cancer, showing IC_50_ equal to 0.24 μM, which is 36-fold more potent than the cisplatin drug (Figure 3A). It should be mentioned that bathophen was the only cytotoxic ligand in all cell lines tested, showing IC_50_ values ranging from 1.10 to 2.38 μM. Among the tested cell lines, A2780 ovarian cells were particularly sensitive to Cu­(**1**)–Cu­(**5**). This result is particularly noteworthy with respect to the cytotoxicity activity against the A2780 cell line, especially in contrast with previously reported related systems, such as the Cu­(phen)_2_(OH)_2_(ClO_4_)_2_ complex. In this context, the complex Cu (**2**) exhibits approximately a 2-fold increase in cytotoxic potency.^40^ Lower selectivity indexes were obtained (SI: 0.86–2.26) since they also affected noncancerous MRC-5 cells (Figure 3B). Due to these promising results, the complex Cu­(**2**) was selected for detailed biological studies.

**Table 3:** In Vitro Cytotoxicity (IC_50_, μM) on A2780 (Ovarian), A549 (Lung), and MCF-7 (Breast) Cancer Cells, and Noncancerous MRC5 Cells after 48 h of Incubation^a^

	A2780	A549	MCF7	MRC5
Cu(**1**)	0.77 ± 0.10	5.43 ± 0.47	1.72 ± 0.14	1.23 ± 0.28
Cu(**2**)	0.24 ± 0.02	1.39 ± 0.14	0.66 ± 0.03	0.42 ± 0.03
Cu(**3**)	9.81 ± 0.11	18.82 ± 0.60	8.63 ± 0.45	8.43 ± 0.36
Cu(**4**)	1.13 ± 0.05	9.38 ± 0.38	7.70 ± 0.43	2.56 ± 0.19
Cu(**5**)	0.59 ± 0.06	5.03 ± 0.52	3.66 ± 0.2	1.20 ± 0.30
phen	>50	>50	>50	>50
bathophen	1.29 ± 0.26	1.10 ± 0.09	2.38 ± 0.16	1.97 ± 0.18
bipy	>50	>50	>50	>50
5,5′-bipy	>50	>50	>50	>50
*tert*-bipy	>50	>50	>50	7.80 ± 1.55
5-Cl2HBz	23.43 ± 0.40	14.37 ± 0.17	14.76 ± 0.47	18.83 ± 0.40
Cu(NO_3_)_2_	21.37 ± 0.35	>50	11.88 ± 0.22	>50
**cisplatin**	8.73 ± 0.45	13.27 ± 0.87	13.98 ± 0.40	29.09 ± 0.78

a Results are presented as a mean ± SD of three independent replicates of IC_50_.

**Figure 3:**
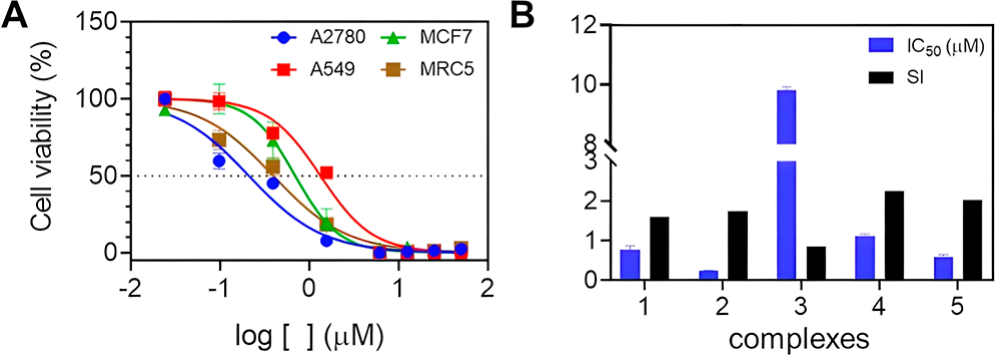
(A) Cell viability (A2780, A549, MCF7, and MRC5) after treatment with complex Cu­(**2**) during 48 h. (B) IC_50_ (μM, A2780) and SI obtained for copper complexes. SI = IC_50_ (MRC5)/IC_50_ (A2780).

The morphology of the A2780 cells was studied upon treatment with Cu­(**2**) at different concentrations (0.12–0.48 μM). As presented in Figure 4, a decrease in cell density and adhesion was observed in the presence of the complex. Significant changes were detected mainly at higher concentrations. Hoechst/PI double staining was used to confirm cell damage. In the absence of Cu­(**2**), only Hoechst fluorescence is detected and, as expected, no cell death was observed. On the other hand, in the presence of the complex, the population of damaged cells increased in a dependent manner, indicating cell death (Figure 4A).

**Figure 4:**
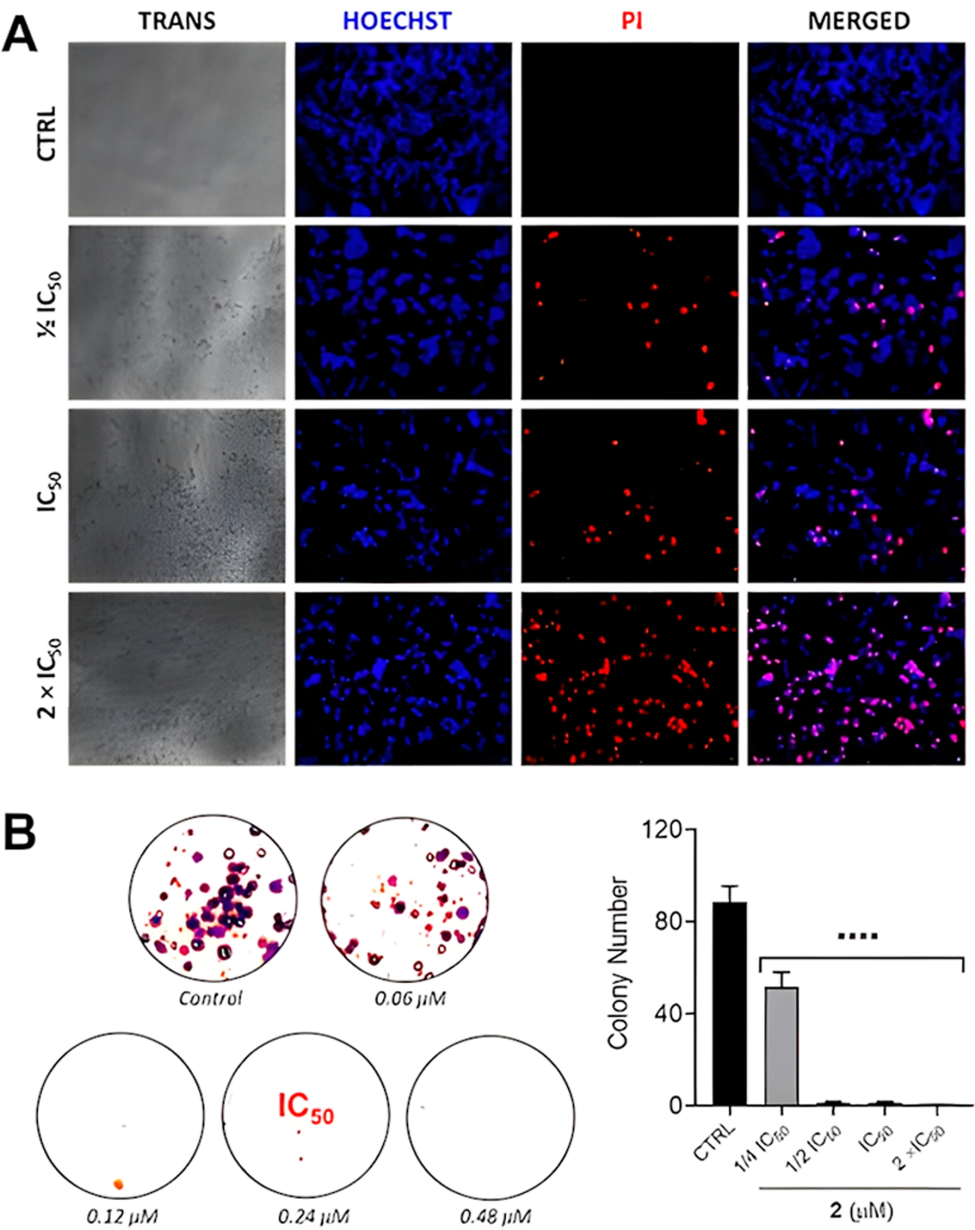
(A) Microscopy images of A2780 cells after 0 and 48 h in the absence (CTRL) and presence of Cu­(**2**) at 1/2 IC_50_, IC_50_, and 2 × IC_50_ concentrations (0.12, 0.24, and 0.48 μM, respectively). For fluorescence images, the cells were treated with Hoechst 33258 and propidium iodide (PI). The negative controls were treated with the DMSO vehicle (0.5% v/v). (B) Representative colony formation images of A2780 cells after treatment with different concentrations of Cu­(**2**). The study was performed in triplicate and the image represents one of them. (D) Quantitative data representing the colony number with relation to the concentration of 2 (*n* = 3, one-way ANOVA test followed by Dunnett’s test). Data are expressed as means ± SD (****P < 0.0001).

In the next step, the antiproliferative potential of Cu­(**2**) was investigated. For this purpose, the clonogenic formation assay was conducted.^41^ The cells were treated with the copper complex at different concentrations (0.06–0.48 μM), and the colony formation was examined over 10 days. Our results revealed that Cu­(**2**) was able to drastically reduce the number of colonies at a concentration of 0.06 and 0.12 μM, which is lower than its IC_50_ value (Figure 4B). These results are in accordance with those reported for different metal-based compounds in ovarian cancer cells.^42–44^

### 3.6 DNA Interacting Properties

DNA is a crucial target for several metal-based compounds.^45^ Considering that copper-based compounds can interact with DNA and the promising results obtained for Cu(2), we decided to investigate its ability to interact with this biomolecule.^46–48^ Several techniques can be used to study different DNA structures.^49,50^ Here, viscosity, circular dichroism (CD), fluorescence, and agarose gel electrophoresis experiments were performed.

First, a viscosity assay was conducted by measuring the relative viscosity of the DNA solution after incubation with the complex. Thiazole orange (TO) and cisplatin were also used as controls. While TO is an intercalating agent that increases the viscosity of the biomolecule, cisplatin acts via covalent bonding, promoting the distortion of the double helix and causing a decrease in viscosity.^51^ As presented in Figure 5A, Cu­(**2**) causes a decrease in the viscosity upon titration with CT-DNA, leading to an analogous trend than cisplatin. In contrast to cisplatin, which is an alkylating agent that binds to the purine bases on the DNA, Cu­(**2**) can act via several ways, and the observed results could be a consequence of different effects, including condensation.^52,53^

**Figure 5:**
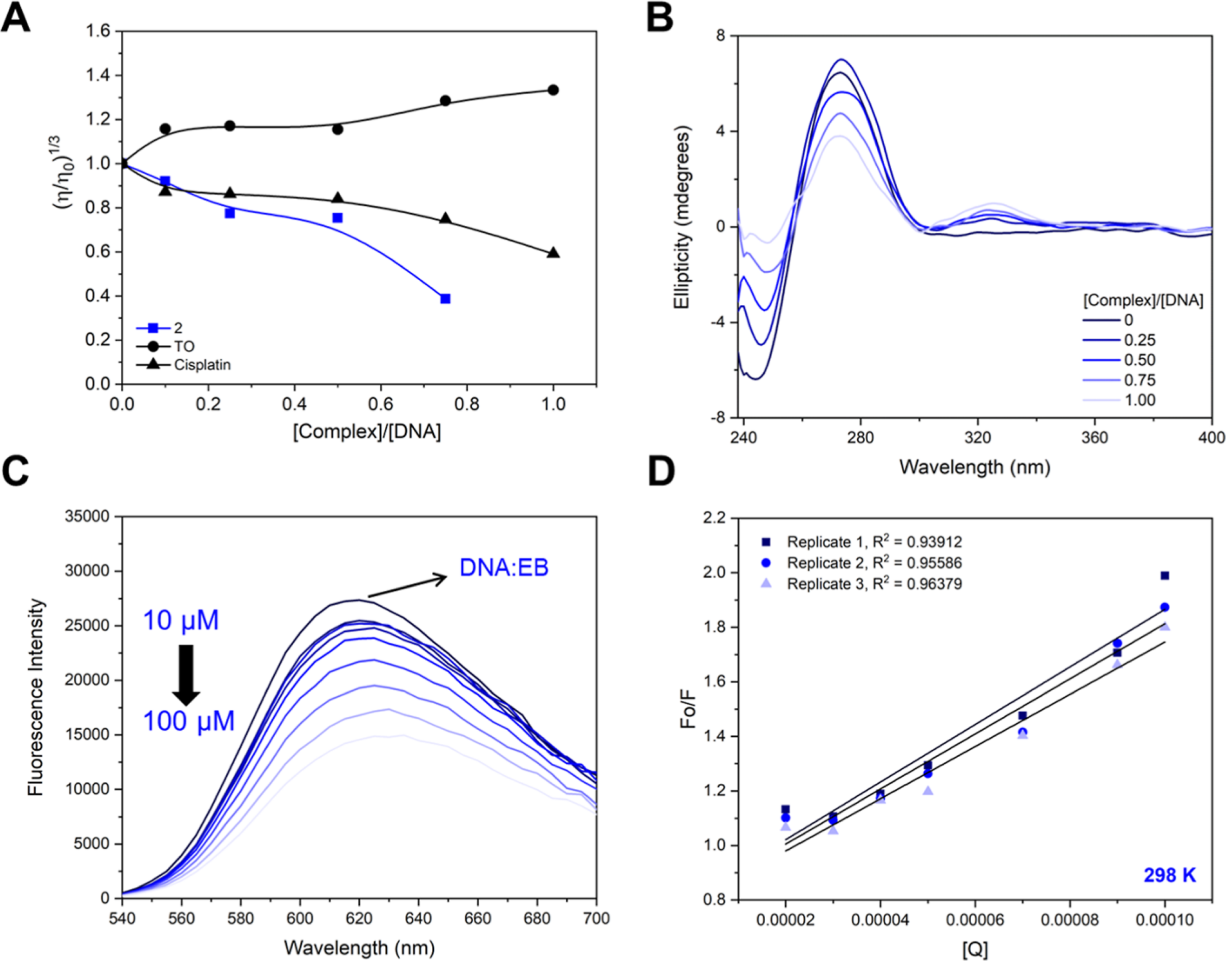
Binding studies with DNA. (A) Effect of increasing concentrations of complex Cu­(**2**), thiazole orange (TO), and cisplatin on the relative viscosity of CT-DNA at 25 °C. (B) CD spectra of CT-DNA (100 μM) in the absence and presence of Cu­(**2**) at different complexes: DNA molar ratios. (C) Fluorescence emission spectra of the EB/DNA complex in the absence and presence of increasing amounts of Cu­(**2**), [EB] = 100 μM, [DNA] = 100 μM, [complex] = 10–100 μM, λexc = 510 nm. (D) Stern–Volmer plot, Q = complex 2.

Subsequently, CD was used to obtain more insights into the DNA/complex interaction. The CD technique is suitable to investigate the structural changes in biomolecules, such as DNA.^54–56^ B-DNA is the most common and studied DNA form, and its CD spectra present two main bands at 275 and 245 nm, which arise from base stacking and right-handed helicity of DNA, respectively. Following an increase in the complex/DNA ratio, the intensity of both bands is affected in a concentration-dependent manner, suggesting significant alterations in the secondary structure of DNA. It is worth noting that such behavior may be associated with DNA condensation and, therefore, should be treated with caution (Figure 5B).^57–59^ Negative band appears to be more sensitive to the complex, and its intensity nearly disappeared at a ratio higher than 0.50. Furthermore, a weak positive signal around 325 nm was observed, which was assigned as an induced CD (ICD) signal arising due to the coupling between the electric transition moments of the complex and the DNA base pairs. These changes are typical of strong interactions between the complex and the biomolecule.^60,61^

Considering the extended aromatic rings of the bathophen ligand, eventually the complex Cu­(**2**) can potentially intercalate into DNA base pairs. We therefore investigated whether complex Cu­(**2**) can interact with DNA through additional modes in addition to covalent ones. For this, we performed a fluorescence competition assay with EB. EB is a DNA-intercalating dye that bounded to DNA emits fluorescence at 610 nm upon excitation at 541 nm. The binding displacement of EB by an external agent causes fluorescence quenching, which may be indicative of an intercalative behavior.^61–63^ Upon an increase in the complex/DNA ratio, the emission decreased as a consequence of fluorescence quenching (Figure 5C). These observations suggest an intercalative contribution to the binding mode.

A quantitative analysis using a Stern–Volmer equation was conducted to evaluate the affinity of complex Cu (**2**) toward CT-DNA. The *K*_SV_ value obtained for Cu (**2**) was 1.01 ± 0.40 × 10^4^ M^–1^, which is lower than those reported for classical intercalators such as EB (10^6^ M^–1^).^64^ However, this value is consistent with results reported for copper­(II)-based compounds and suggests that eventually Cu (**2**) can displace EB and intercalate between the DNA base pairs.

It is worth noting that the displacement leading to fluorescence quenching does not necessarily confirm pure intercalation, since EB can be displaced by various DNA binders.^65,66^ As reported, different binding modes can take place, and due to their structural diversity, several compounds interact through a multimodal binding mechanism ^67^. Using the pBR322 plasmid DNA, the gel electrophoresis experiments were used to investigate the DNA cleavage properties of Cu­(**2**). Three distinct plasmid forms were observed, whose mobility depends on their topology: (i) supercoiled DNA (SC) that migrates faster on the gel, (ii) open circular DNA form (OC), resulting from single-strand scission, which exhibits a slower migration, and (iii) linear form (L), arising from double-strand cleavage, which migrates between SC and OC forms. As expected, two bands are observed for the plasmid (Figure S40, lane 1, see Supporting Information), corresponding to the SC and OC forms. The altered plasmid mobility behavior observed in lane 2 may be attributed to cisplatin-induced DNA cross-linking. In contrast to other copper-based compounds, no plasmid cleavage was detected after incubation with Cu­(**2**) at the maximum concentration tested (lanes 3–8).^61^

## 4 Conclusions

Five novel heteroleptic Cu­(II) complexes with 5-chloro-2-hydroxybenzophenone and N–N donor ligands were synthesized and characterized. Single-crystal X-ray diffraction confirmed distinct coordination geometries, while spectroscopic studies demonstrated stability in solution.

All complexes exhibited cytotoxic activity; however, complex Cu (**2**) displayed potent cytotoxicity against A2780 ovarian cancer cells (IC_50_ = 0.24 μM), surpassing that of cisplatin by 36-fold. The studies carried out here revealed that the DNA–Cu­(**2**) interaction occurs via multimodal interaction modes, affecting the structure of the double-helix. These findings highlight the potential of Cu­(II)/2-hydroxybenzophenone complexes as tunable anticancer agents, with Cu­(**2**) containing bathophen emerging as a particularly promising candidate for further development.

## Supplementary Material



## Notes

The Supporting Information provides detailed experimental and analytical data for complexes described in the mais text. It includes elemental analysis, thermogravimetric analysis, infrared spectra, Raman spectra, UV–vis spectra, ESI spectra, EPR spectra, stability in solution, and agarose gel electrophoresis experiment. Additionally, single-crystal X-ray diffraction data and crystallographic parameters of all complexes are presented (PDF)
