# Supplementary material for: Copper(II)-5-chloro-2-hydroxybenzophenone Complexes with N–N Donors: Structural Insights and Antitumor Activity against A2780 Human Ovarian Cancer Cells of a Bathophen Derivative
Source: ACS Omega. 2026 Mar 3;11(10):16321–32. doi: 10.1021/acsomega.5c11889 (PMC13000599; doi:10.1021/acsomega.5c11889)
Supplement: Supplementary file 1 [file ao5c11889_si_006.pdf]

Supporting Information

Copper(II)-5-chloro-2-hydroxybenzophenone Complexes with *N-N* Donors: Structural Insights and Antitumor Activity Against A2780 human ovarian cancer cells of a Bathophen Derivative

Alexandre B. de Carvalho[a], *Marcos V. Palmeira-Mello*[b], *Paulo N. de Souza*[a], *Saulo H. Mendes Abe*[b], *José G. Balena Filho*[c], *Marcelo B. Andrade*[d], *Rodrigo S. Corrêa*[d], *Alzir A. Batista*[b], *Javier Ellena*[a*]

[a] *São Carlos Institute of Physics, University of São Paulo, IFSC – USP, 13566-950, São Carlos, SP, Brazil*

[b] *Departament of Chemistry, Federal University of São Carlos (UFSCar), 13565-905 São Carlos, São Paulo, Brazil.*

[c] *Nanotechnology National Laboratory for Agriculture (LNNA), EMBRAPA Instrumentation, 13561-206 São Carlos, São Paulo, Brazil.*

[d] *Departament of Physics, Institute of Exact and Biological Sciences, Federal University of Ouro Preto (UFOP), 35402-136 Ouro Preto, Minas Gerais, Brazil.*

[e] *Departament of Chemistry, Institute of Exact and Biological Sciences, Federal University of Ouro Preto (UFOP), 35402-136 Ouro Preto, Minas Gerais, Brazil.*

[a*] Corresponding author

email: javiere@if.sc.usp.br

Table of Contents

Part I – Thermogravimetric Analysis……………………………………………….1

Part II – IR Spectra………………………………………………………………..3

Part III – Raman Spectra……………………………………………………………6

Part IV- UV-vis Spectra……………………………………………………….……..9

Part V- ESI-MS Spectra……………………………………………………....12

Part VI- Crystallographic data…………………………………………………..15

Part VII- EPR Spectra……………………………………………………………...20

Part VIII- Stability in solution………………………………………………..25

Part IX- Electrophoresis…………………………………………………………...26

Part I- Thermogravimetric Analysis

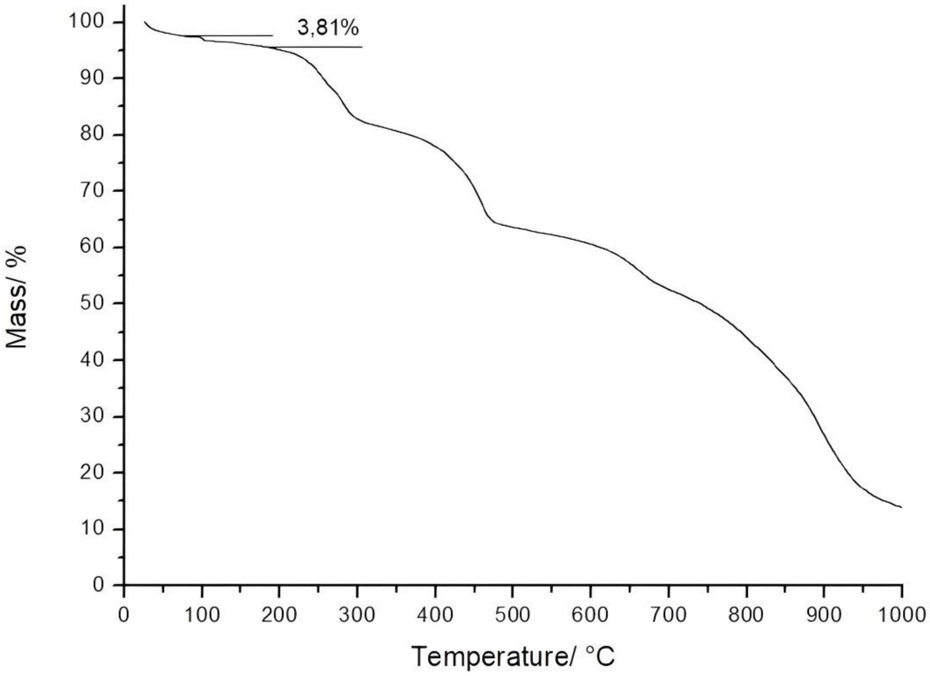

Figure S1. Thermogravimetric curve of Cu (**2**). The experiment was carried out in an N_2 atmosphere, with a flow rate of 50 mL min^{-1} and a heating rate of 10 °C min^{-1}. The temperature range was 10 to 1000 °C.

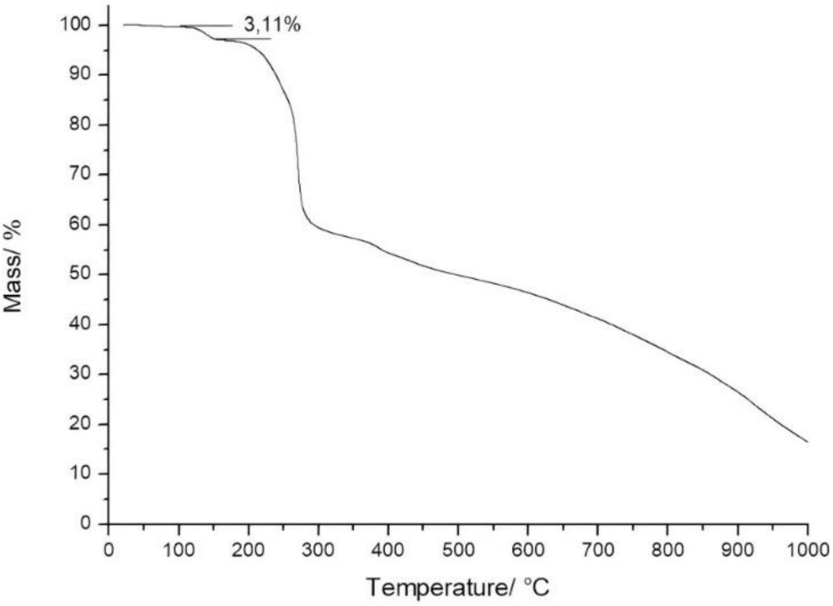

Figure S2. Thermogravimetric curve of Cu (**3**). The experiment was carried out in an N_2 atmosphere, with a flow rate of 50 mL min^{-1} and a heating rate of 10 °C min^{-1}. The temperature range was 10 to 1000 °C.

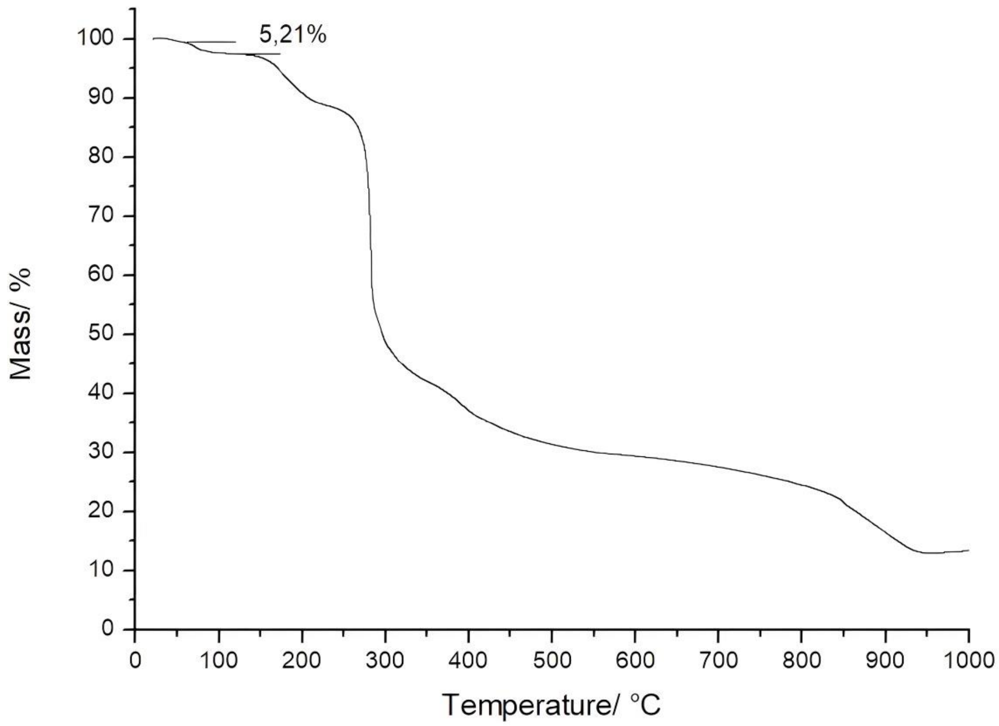

Figure S3. Thermogravimetric curve of Cu (**5**). The experiment was carried out in an N_2 atmosphere, with a flow rate of 50 mL min^{-1} and a heating rate of 10 °C min^{-1}. The temperature range was 10 to 1000 °C.

Part II- IR spectra

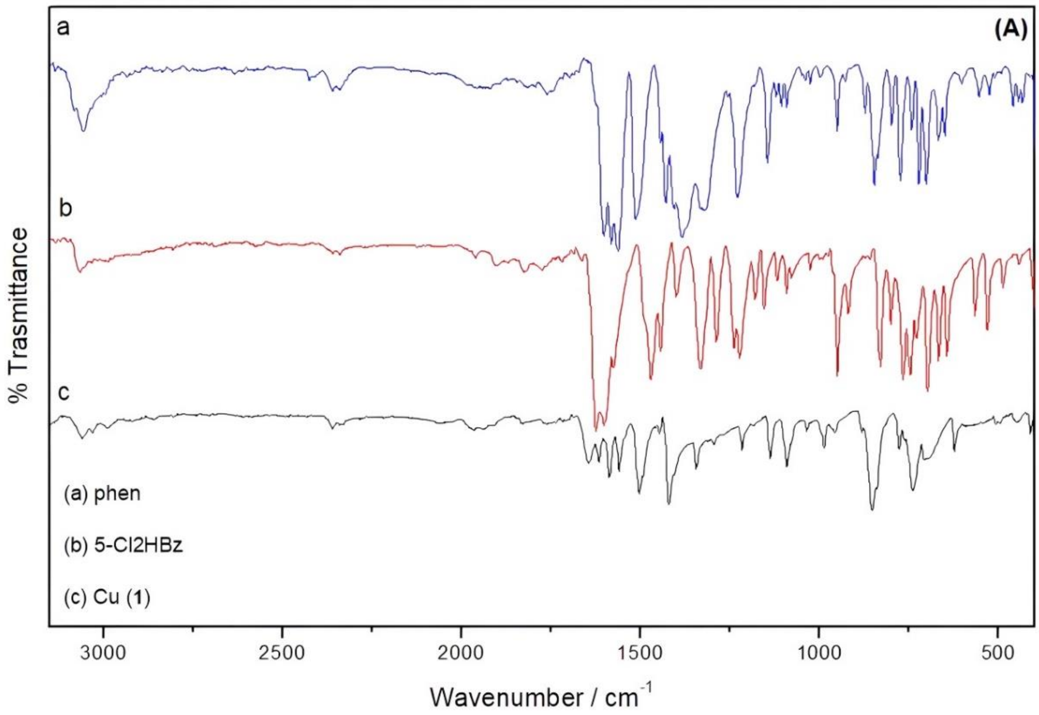

Figure S4. IR spectra of the complex Cu (**1**), in KBr.

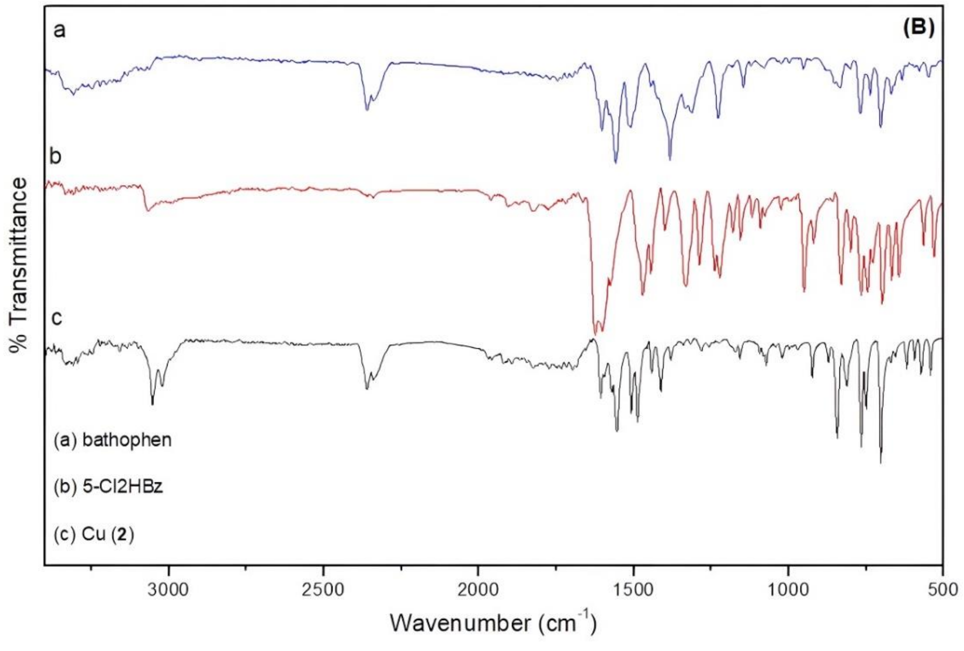

Figure S5. IR spectra of the complex Cu (**2**), in KBr.

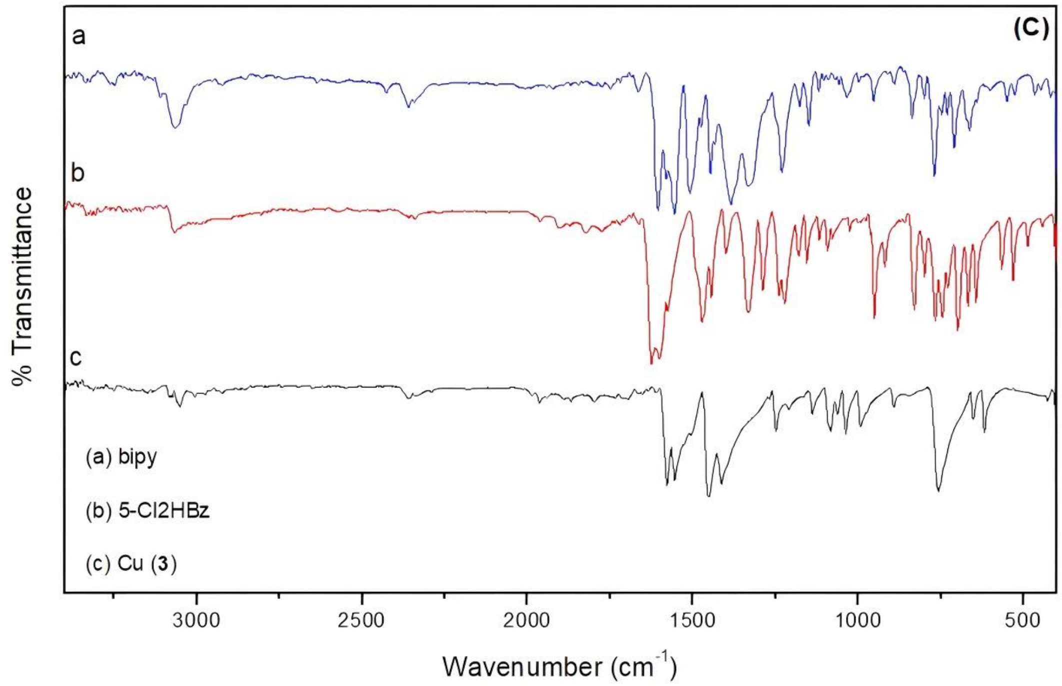

Figure S6. IR spectra of the complex Cu (**3**), in KBr.

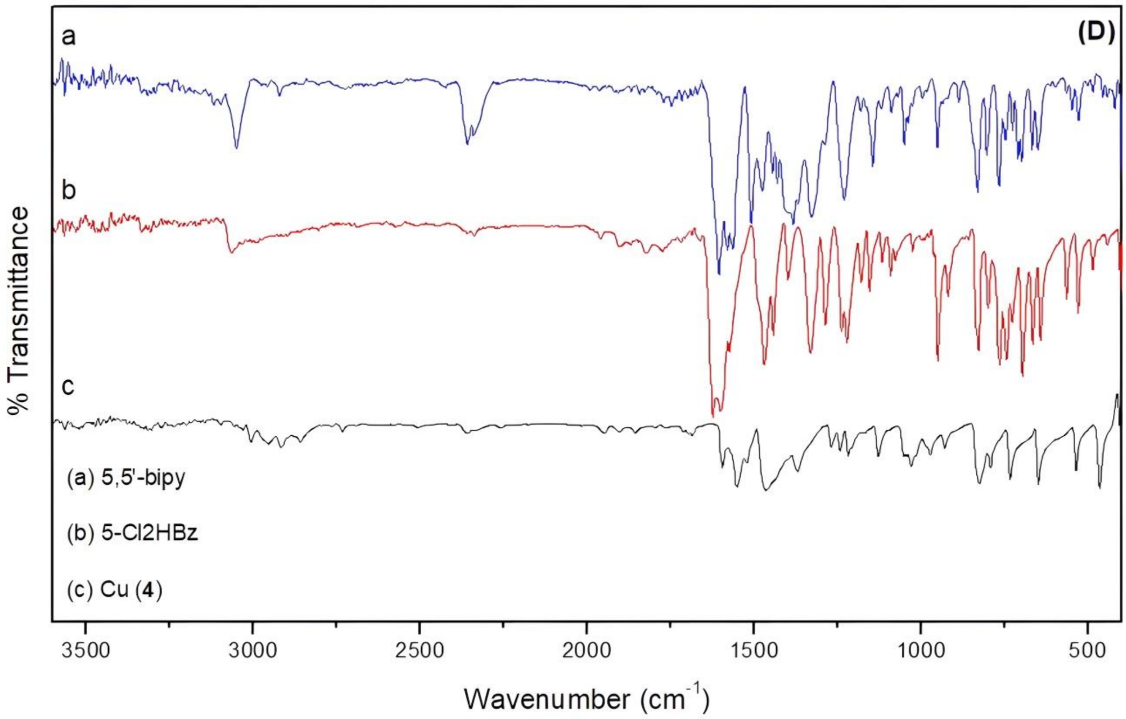

Figure S7. IR spectra of the complex Cu (**4**), in KBr.

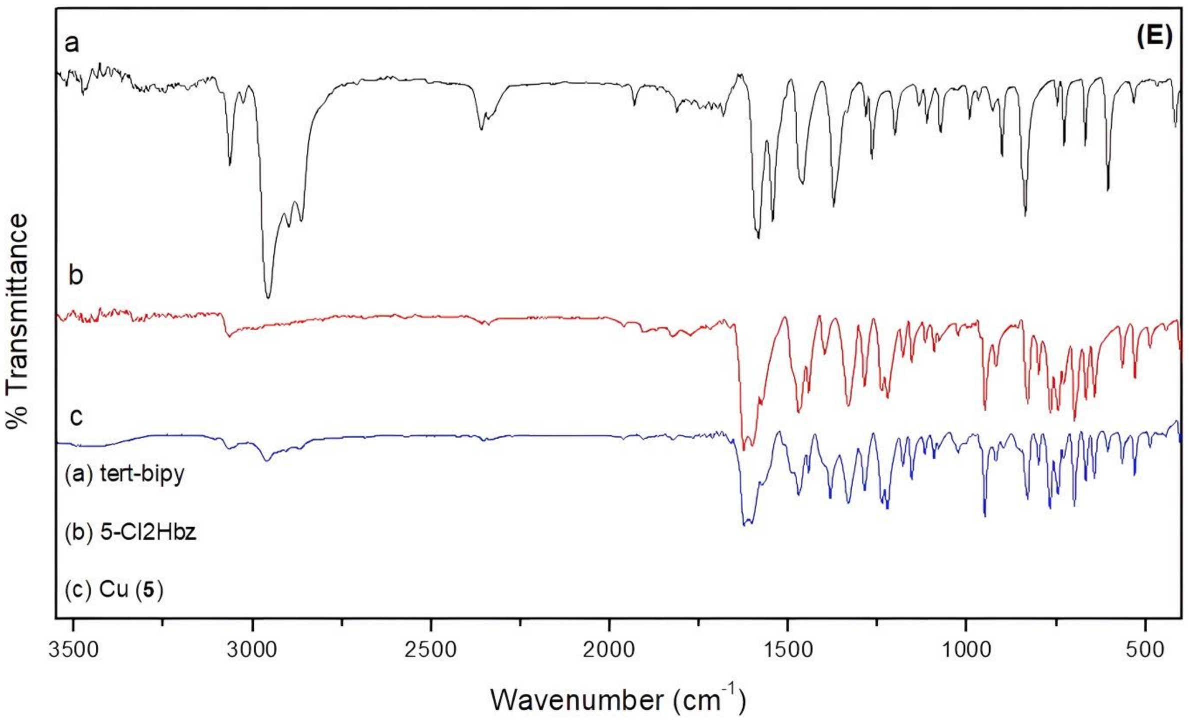

Figure S8. IR spectra of the complex Cu (**5**), in KBr.

Part III- RAMAN spectra

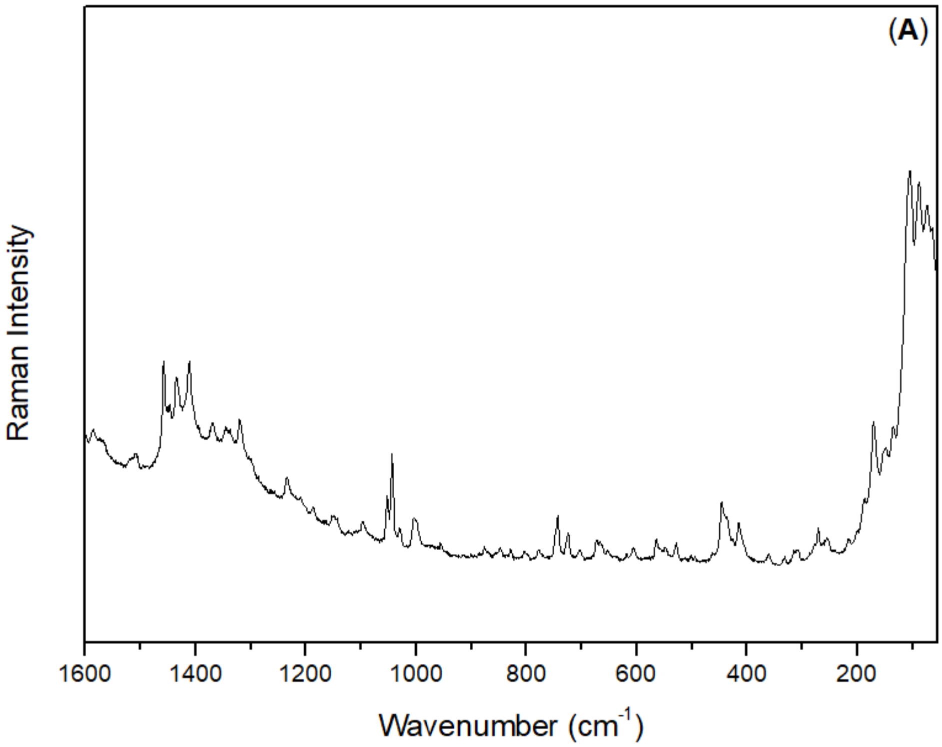

Figure S9. Raman spectra of the complex Cu (**1**).

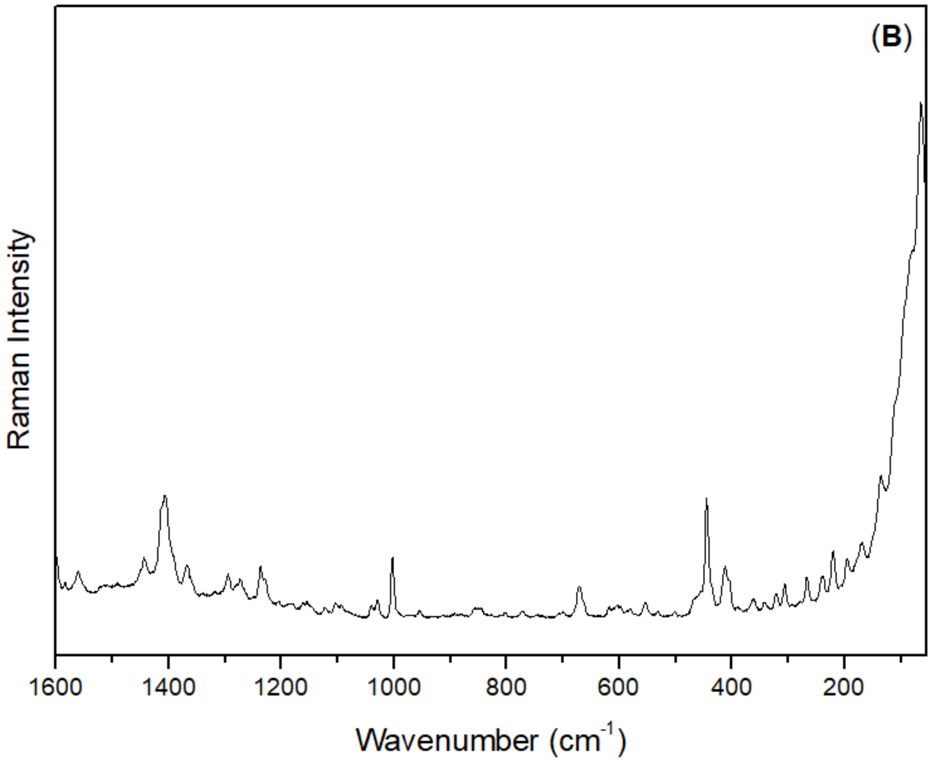

Figure S10. Raman spectra of the complex Cu (**2**).

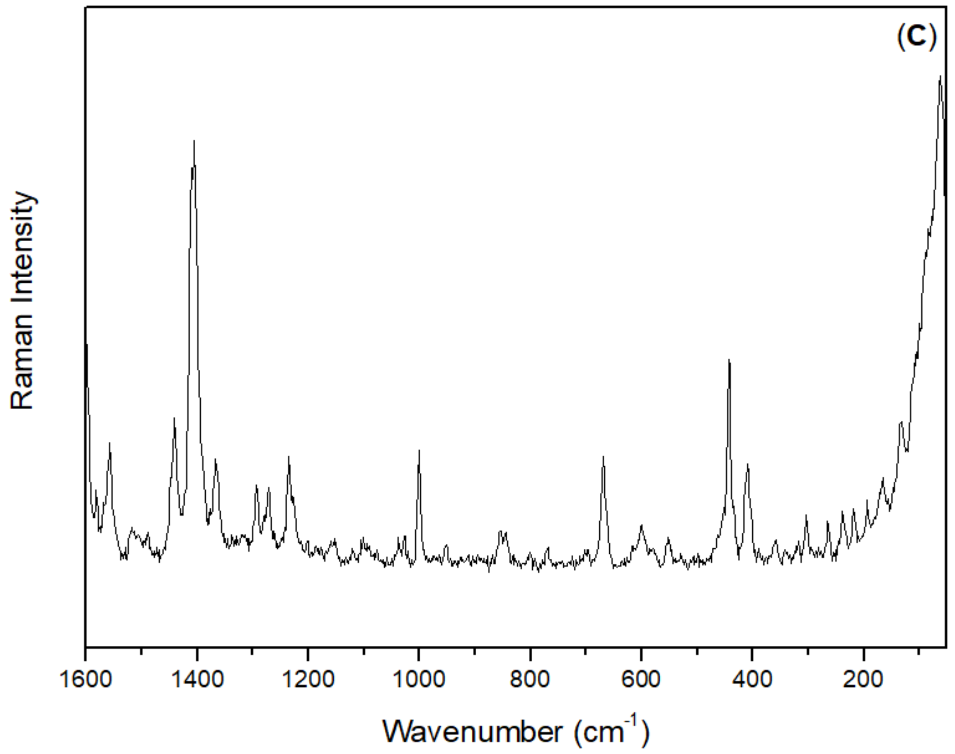

Figure S11. Raman spectra of the complex Cu (**3**).

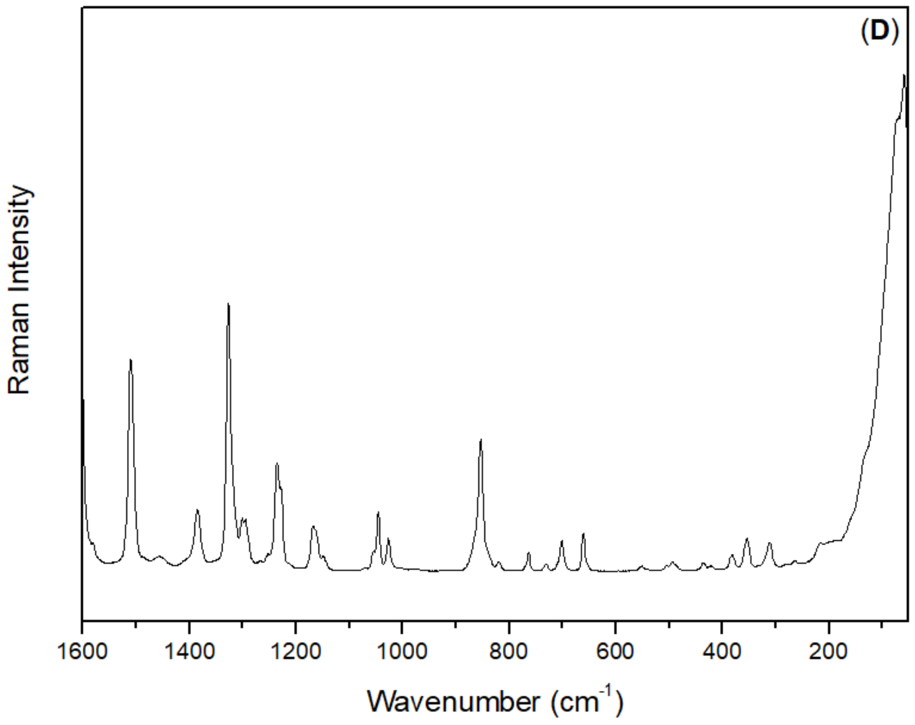

Figure S12. Raman spectra of the complex Cu (**4**).

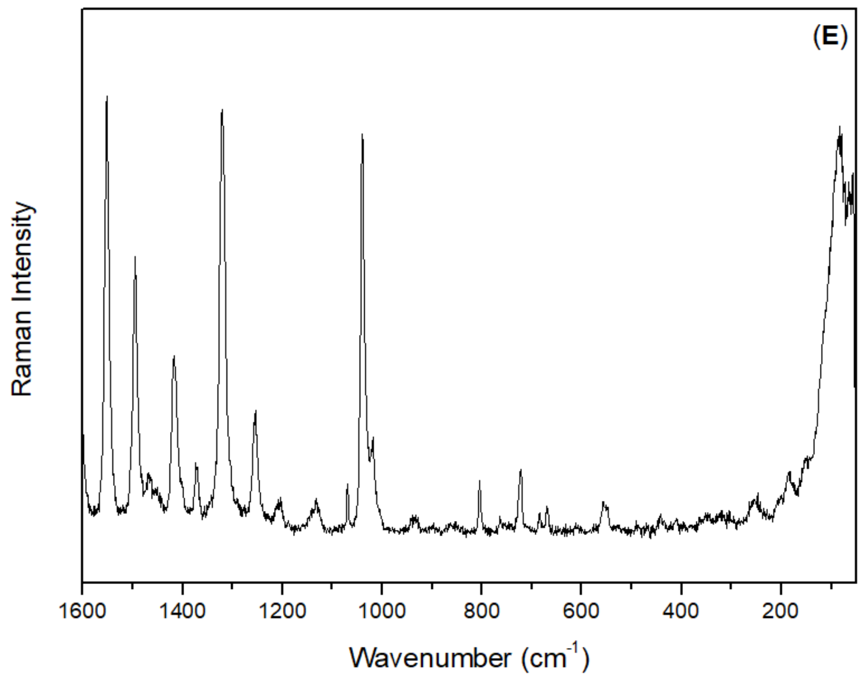

Figure S13. Raman spectra of the complex Cu (**5**).

Part IV- UV/vis spectra

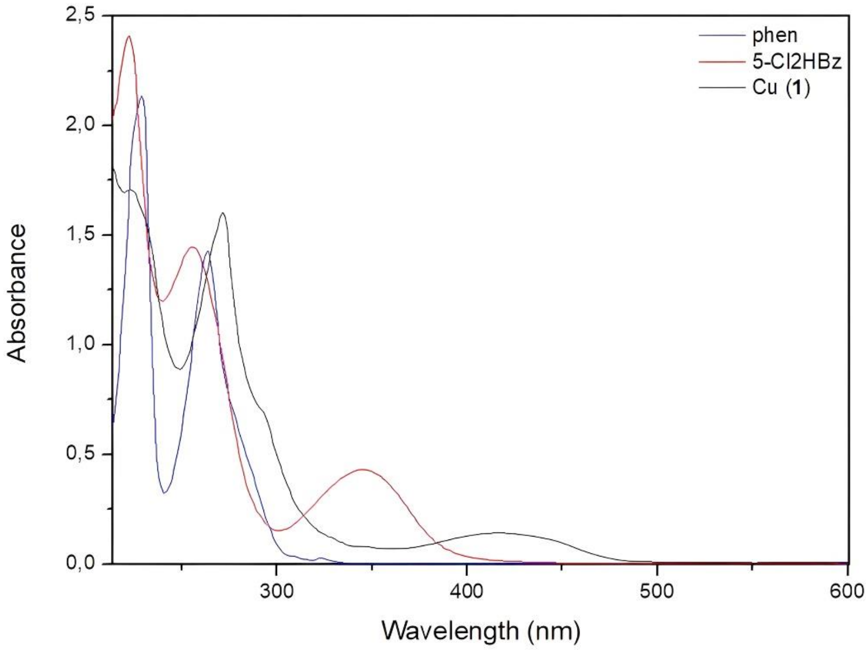

Figure S14. Uv-vis spectra of phen, 5-Cl2HBz and Cu (**1**) in methanol at 10^{-3} M.

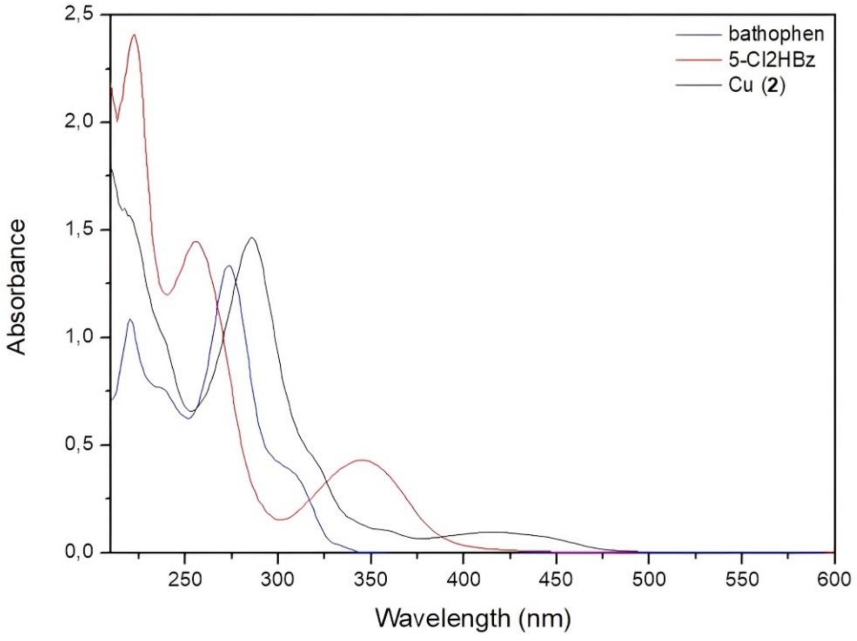

Figure S15. Uv-vis spectra of bathophen, 5-Cl2HBz and Cu (**2**) in methanol at 10^{-3} M.

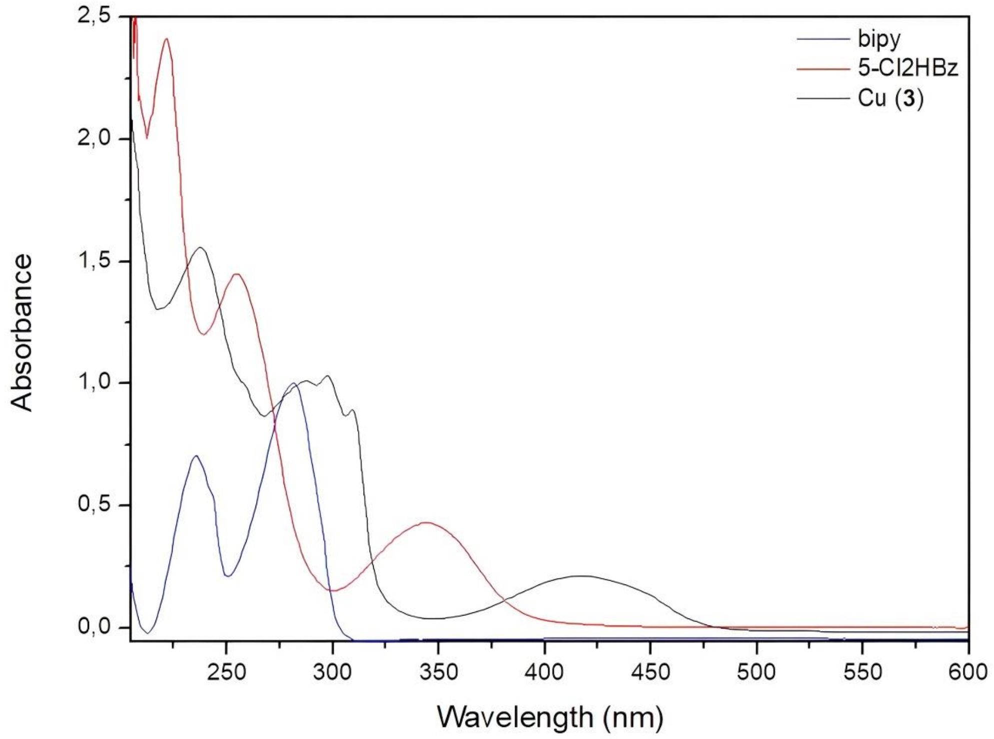

Figure S16. Uv-vis spectra of bipy, 5-Cl2HBz and Cu (**3**) in methanol at 10^{-3}.

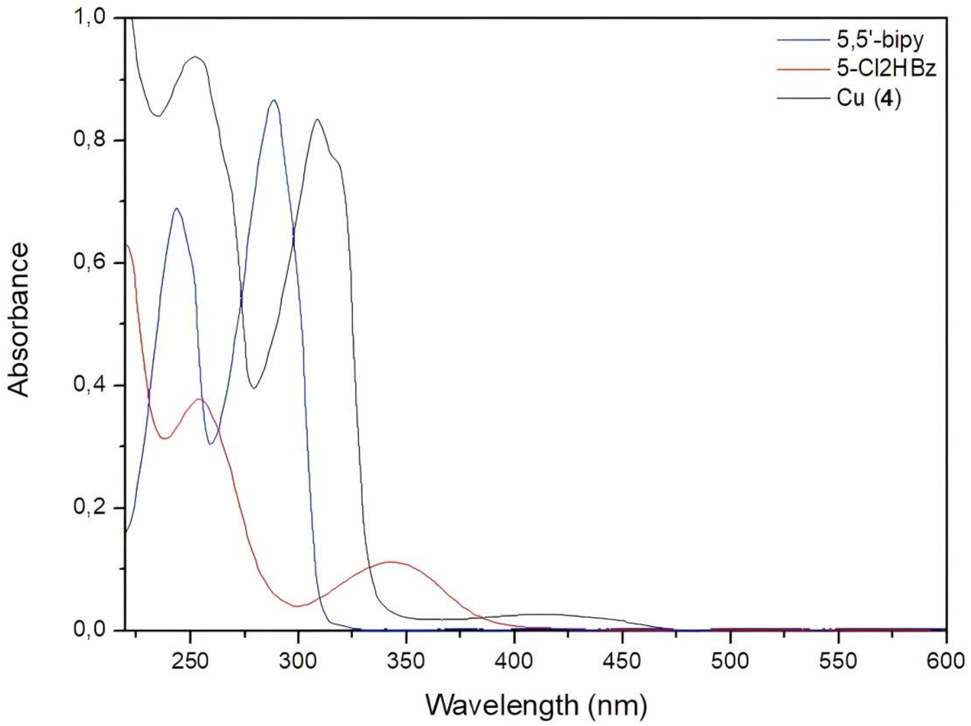

Figure S17. Uv-vis spectra of 5,5'-bipy, 5-Cl2HBz and Cu (**4**) in methanol at 10^{-3} M.

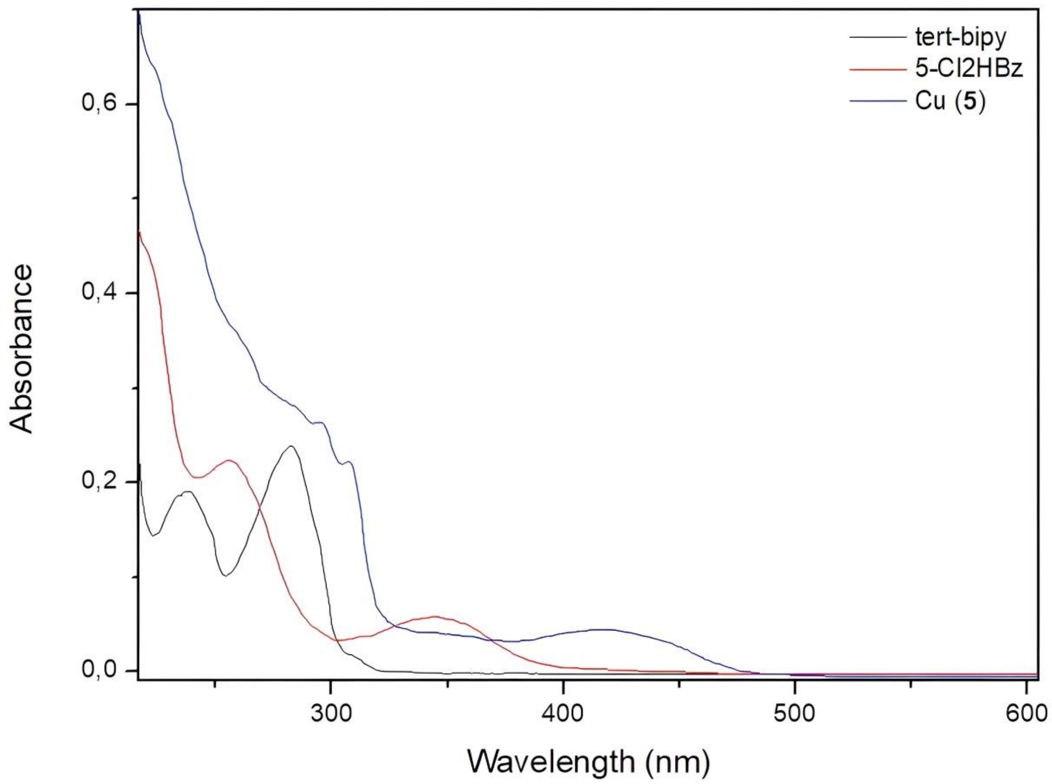

Figure S18. Uv-vis spectra of tert-bipy, 5-Cl2HBz and Cu (**5**) in methanol at 10^{-3} M.

Part V- ESI-MS

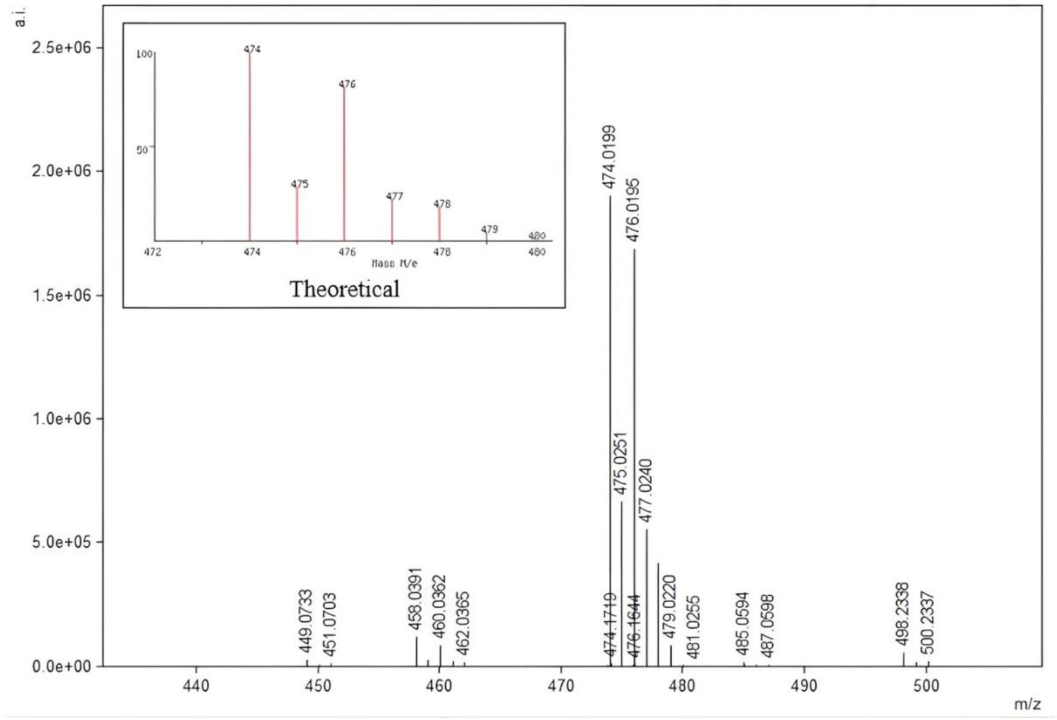

Figure S19. ESI/MS spectrum of Cu (**1**) in methanol

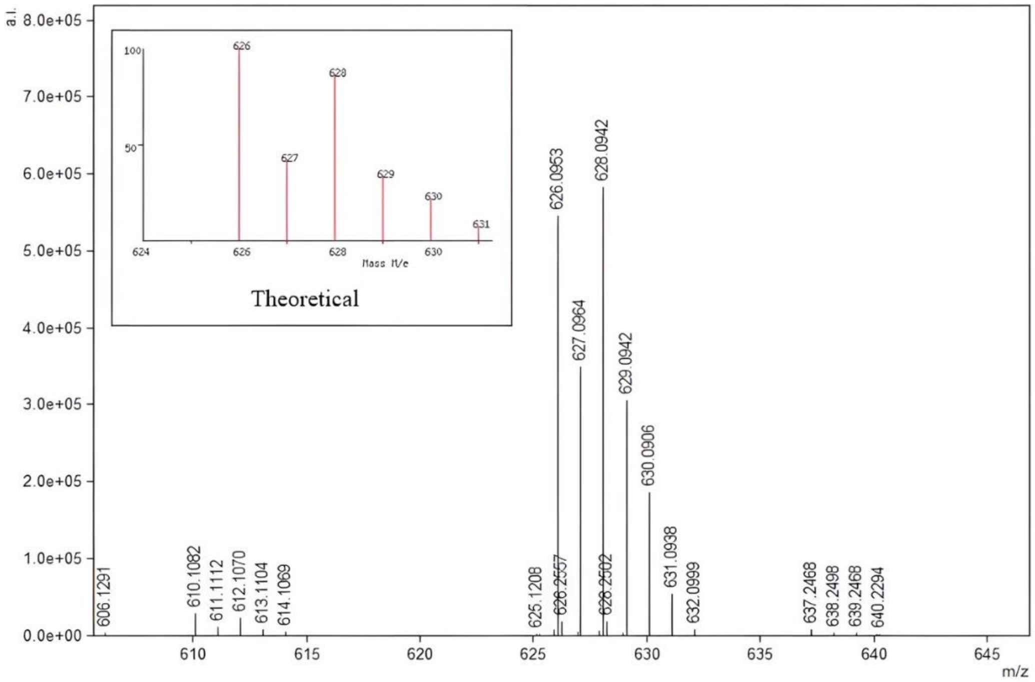

Figure S20. Mass spectrum of Cu (**2**) in methanol.

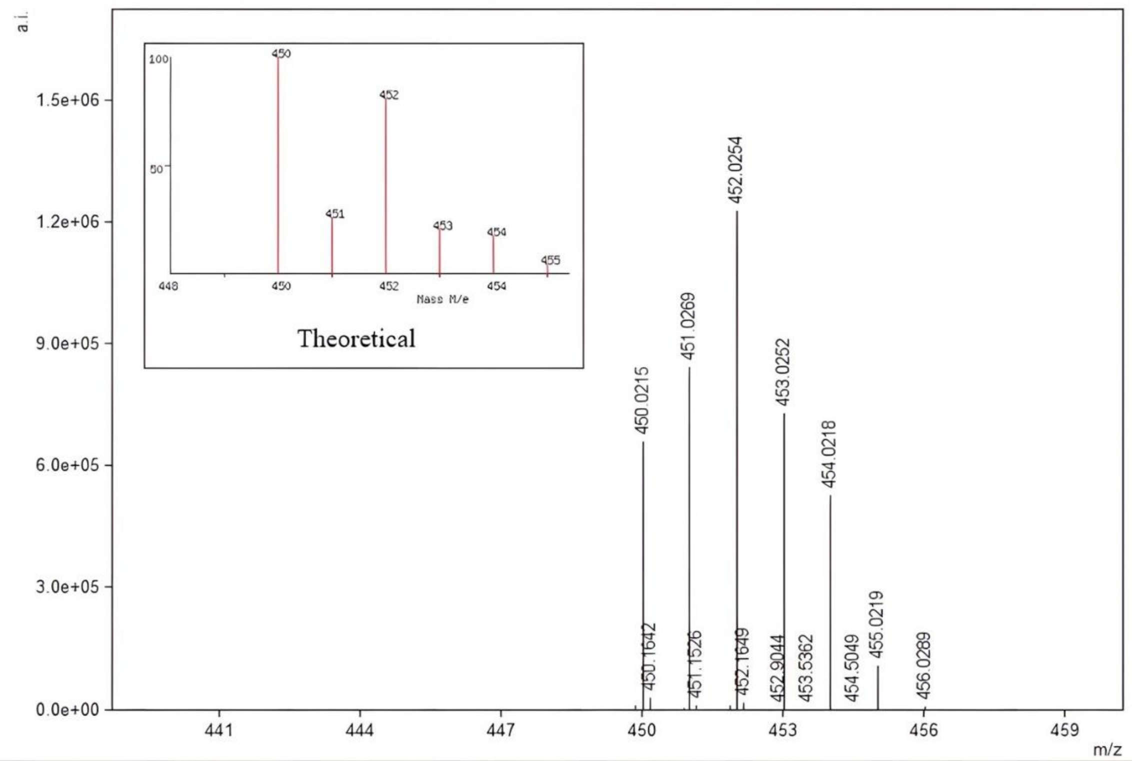

Figure S21. Mass spectrum of Cu (**3**) in methanol.

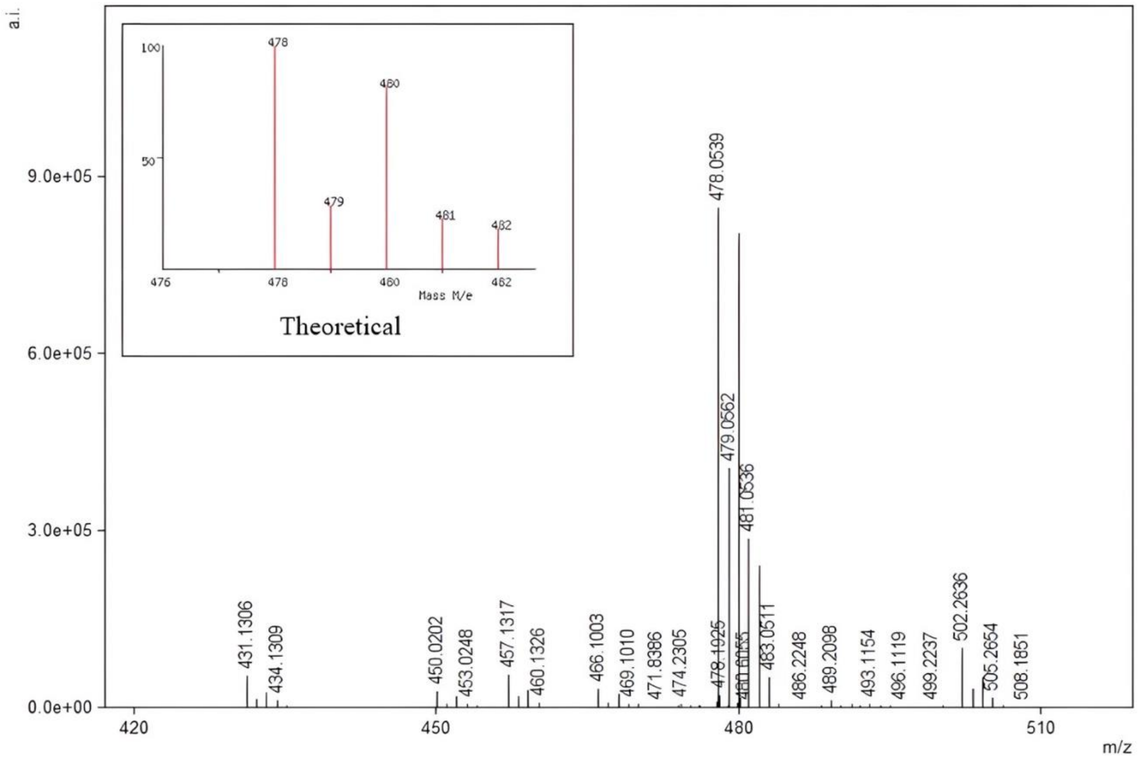

Figure S22. Mass spectrum of Cu (**4**) in methanol.

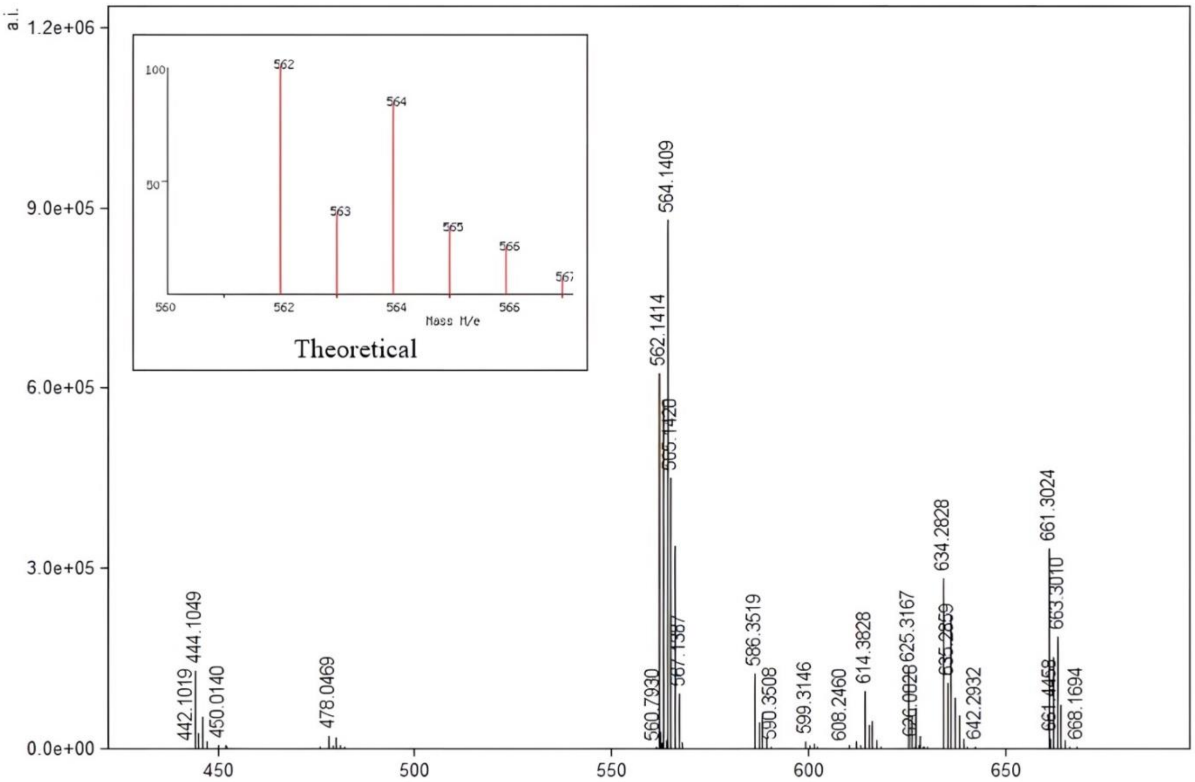

Figure S23. Mass spectrum of Cu (**5**) in methanol

Part VI- Crystallographic data

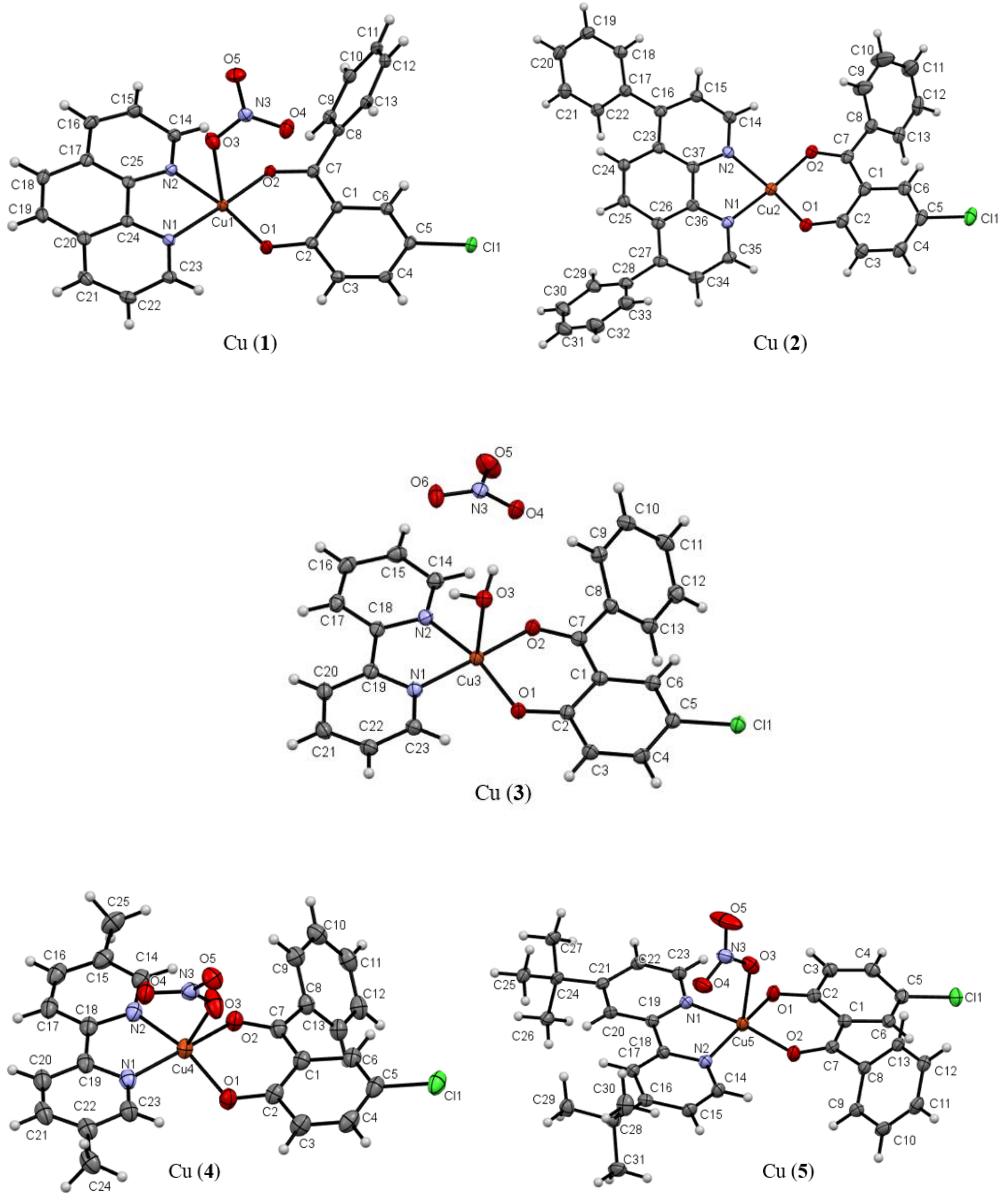

Figure S24. ORTEP type view and atomic numbering of Cu (**1-5**), showing atoms labelling scheme and 50% probability ellipsoids. for Cu (**2**) the counterion NO3- and the solventeH2O and CH3OH were omitted for clarity

Table S1. The bond distance and angles of Cu (**1-5**)

	Cu (**1**)	Cu (**2**)*	Cu (**3**)	Cu (**4**)	Cu (**5**)
	Bond Distance (Å)				
Cu-O1	1.8808 (12)	1.8818 (12)	1.8859 (11)	1.9255 (14)	1.8977 (12)
Cu-O2	1.9280 (12)	1.9518 (12)	1.9417 (11)	1.9536 (13)	1.9292 (12)
Cu-O3	2.4220 (14)	--	2.3381 (11)	2.2352 (16)	2.3754 (13)
Cu-N1	2.0005 (15)	1.9972 (14)	1.9906 (13)	1.9847 (16)	1.9843 (13)
Cu-N2	1.9986 (14)	2.0072 (14)	1.9920 (14)	2.0039 (16)	1.9871 (16)
	Bond Angle (°)				
O1-Cu-O2	92.88 (5)	92.00 (5)	91.52 (5)	90.41 (6)	92.18 (5)
O2-Cu-O3	91.82 (5)	--	91.95 (5)	87.62 (6)	95.20 (5)
O1-Cu-O3	107.94 (5)	--	101.32 (5)	88.80 (7)	90.46 (5)
O1-Cu-N2	171.17 (6)	167.26 (5)	163.85 (5)	161.81 (7)	169.03 (6)
N1-Cu-O3	91.17 (5)	--	93.90 (5)	102.80 (6)	91.69 (5)
N2-Cu-O3	79.26 (5)	--	93.90 (5)	109.37 (7)	99.44 (5)
N2-Cu-O2	173.09 (5)	94.87 (5)	92.87 (5)	90.99 (6)	91.62 (5)
N1-Cu-O2	91.98 (6)	170.69 (5)	172.22 (5)	168.77 (6)	171.42 (5)
N1-Cu-O1	92.12 (6)	89.62 (5)	92.34 (5)	93.94 (6)	92.86 (5)
N1-Cu-N2	82.47 (6)	81.92 (6)	81.62 (6)	81.68 (7)	82.20 (6)

* This complex exhibit 1.5 water molecule and one molecule of methanol in solvent mask.

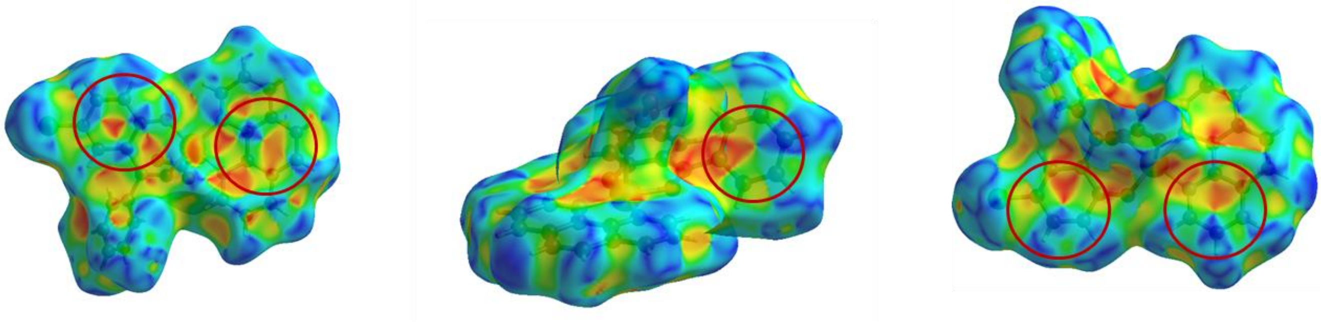

Figure S25. Detail Hirshfeld surfaces mapped in shape index for Cu (**1**) complex.

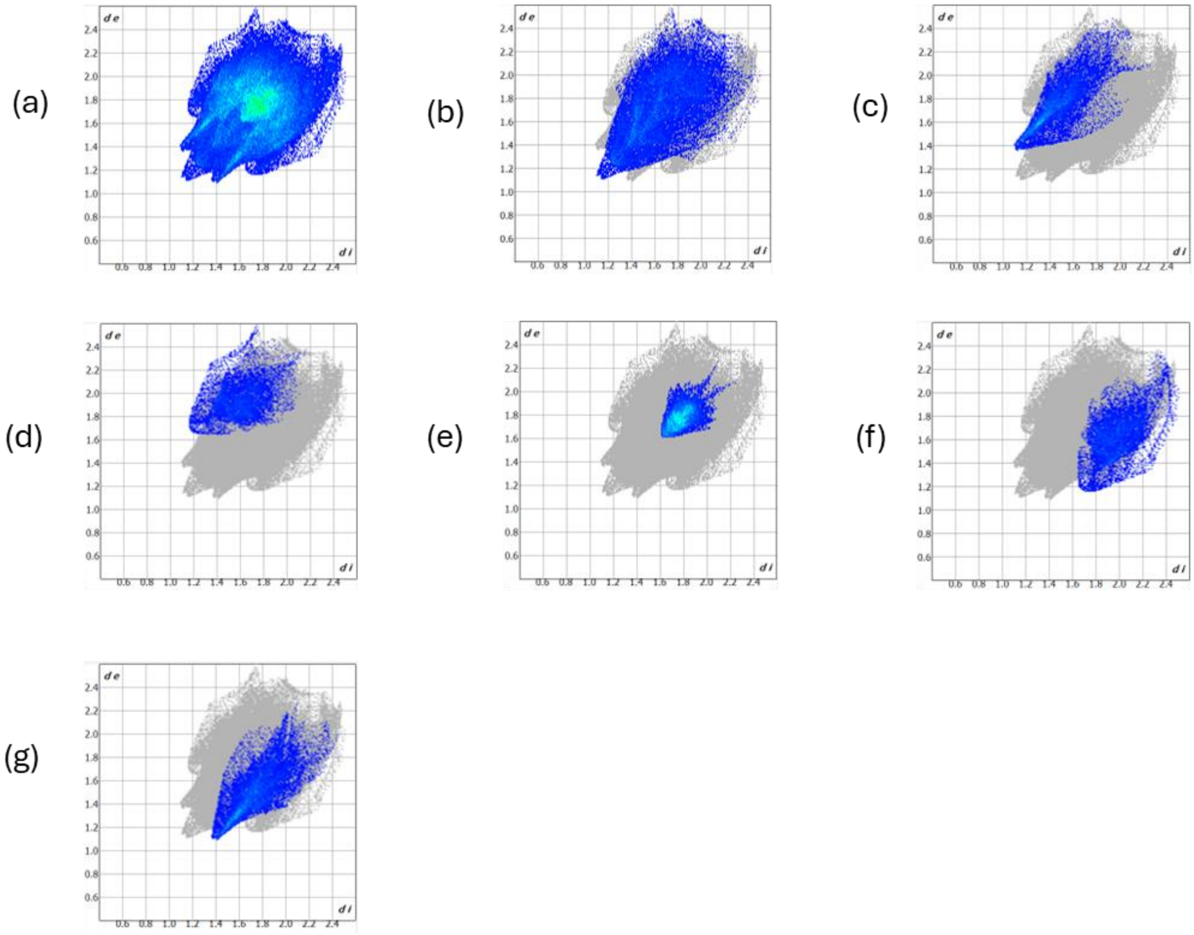

Figure S26. Fingerprint images of (a) Total, (b) H-H, (c) H-O, (d) H-C, (e) C-C, (f) C-H and (g) O-H of Cu (**1**).

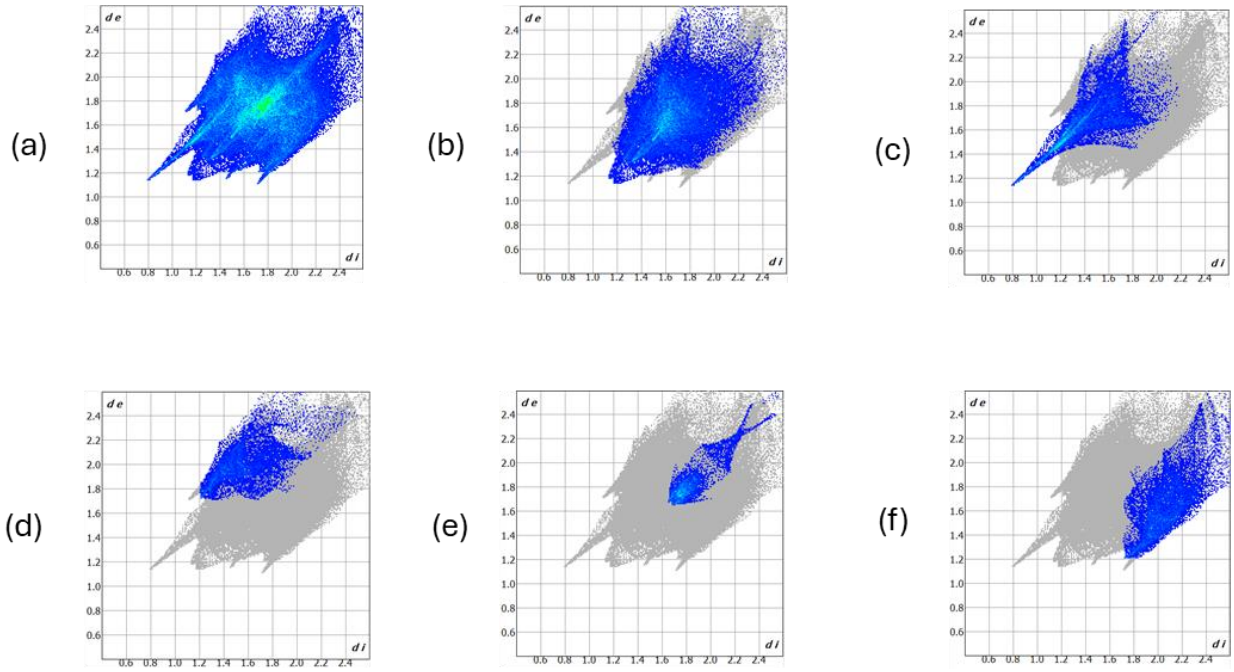

Figure S27. Fingerprint images of (a) Total, (b) H-H, (c) H-O, (d) H-C, (e) C-C and (f) C-H of Cu (**3**).

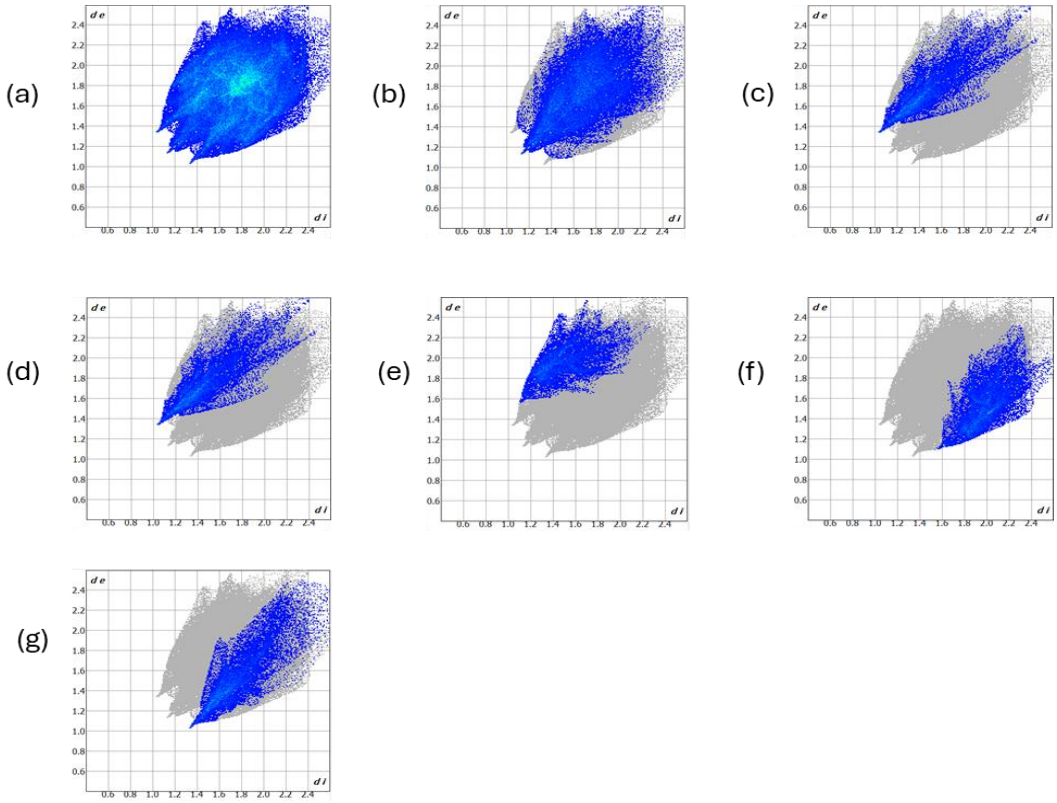

Figure S28. Fingerprint images of (a) Total, (b) H-H, (c) H-O, (d) H-C, (e) C-C, (f) C-H and (g) O-H of Cu (**4**).

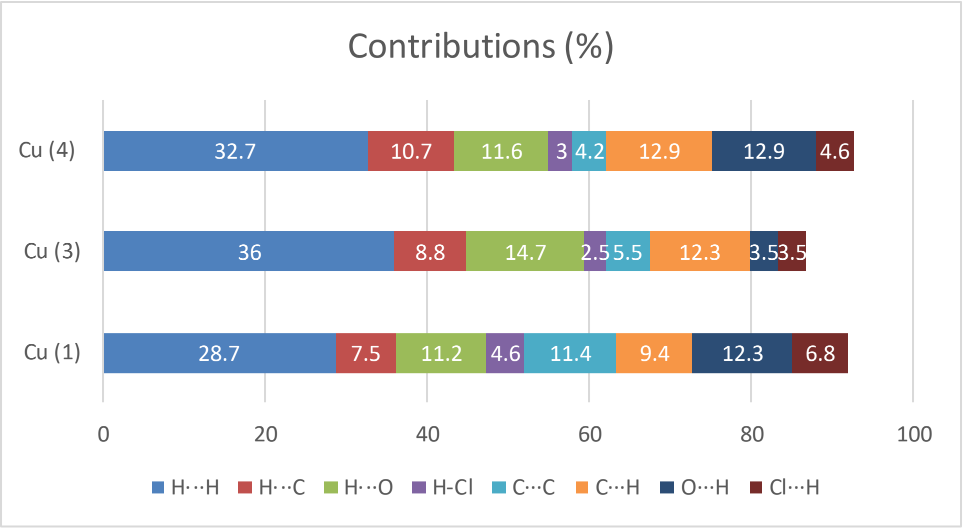

Figure S29. Contributions (%) of the contacts for the structure of the Cu (**1**), Cu (**3**) and Cu (**4**) complexes.

Part VII- EPR spectra

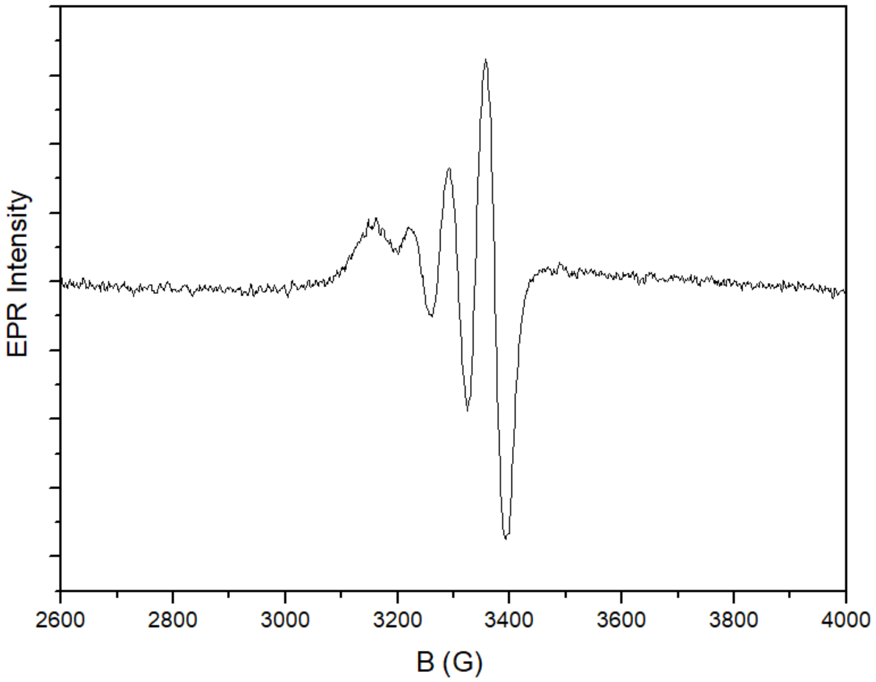

Figure S30. EPR spectrum of the complex Cu (**1**) at 10mM in methanol.

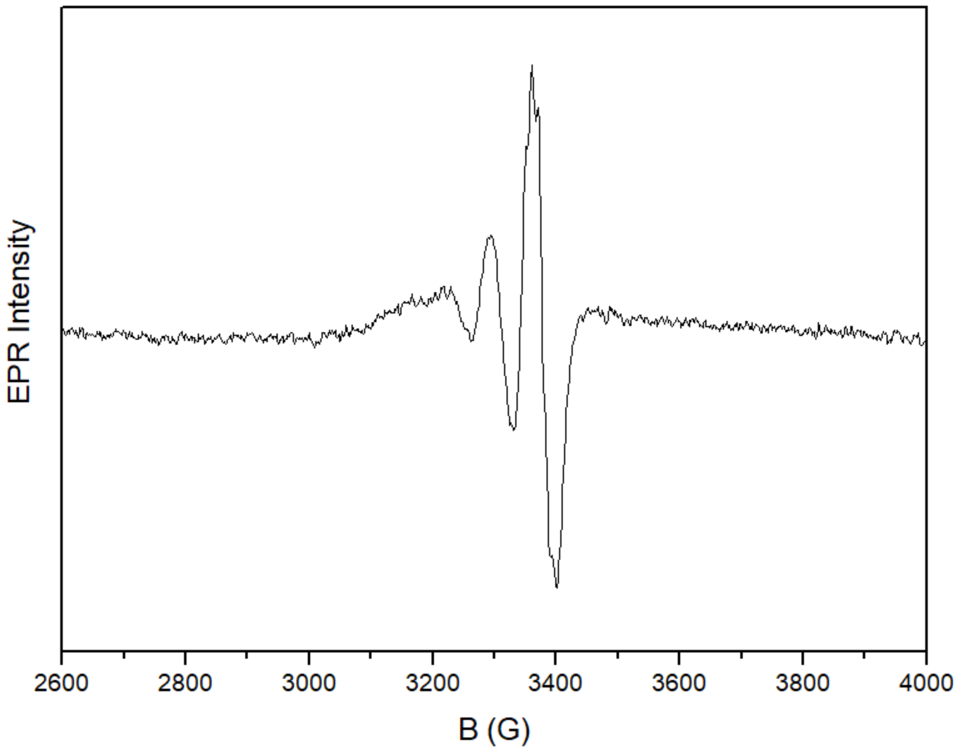

Figure S31. EPR spectrum of the complex Cu (**2**) at 10mM in methanol.

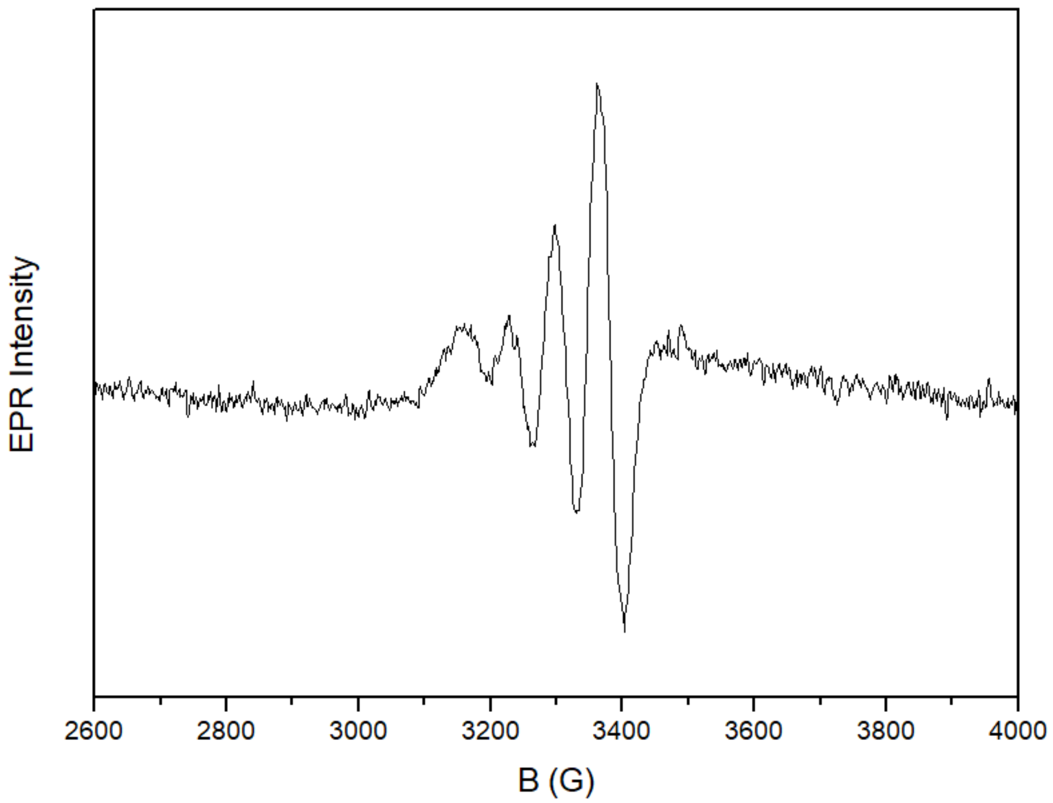

Figure S32. EPR spectrum of the complex Cu (**3**) at 10mM in methanol.

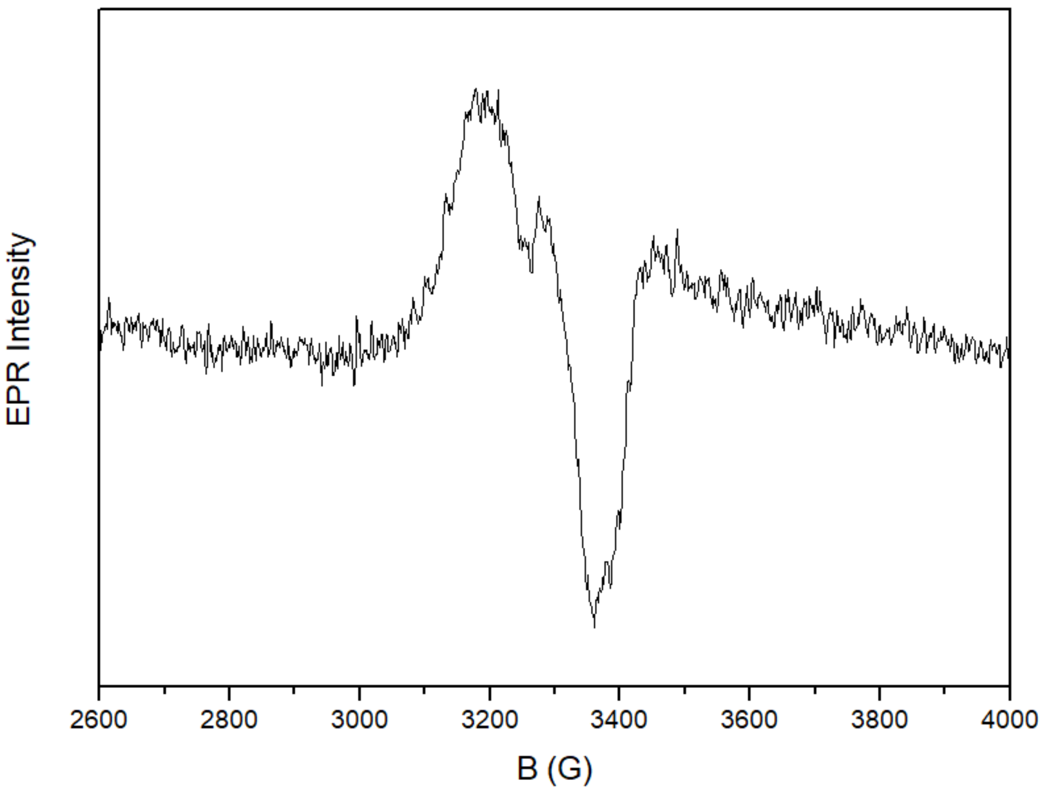

Figure S33. EPR spectrum of the complex Cu (**4**) at 10mM in methanol.

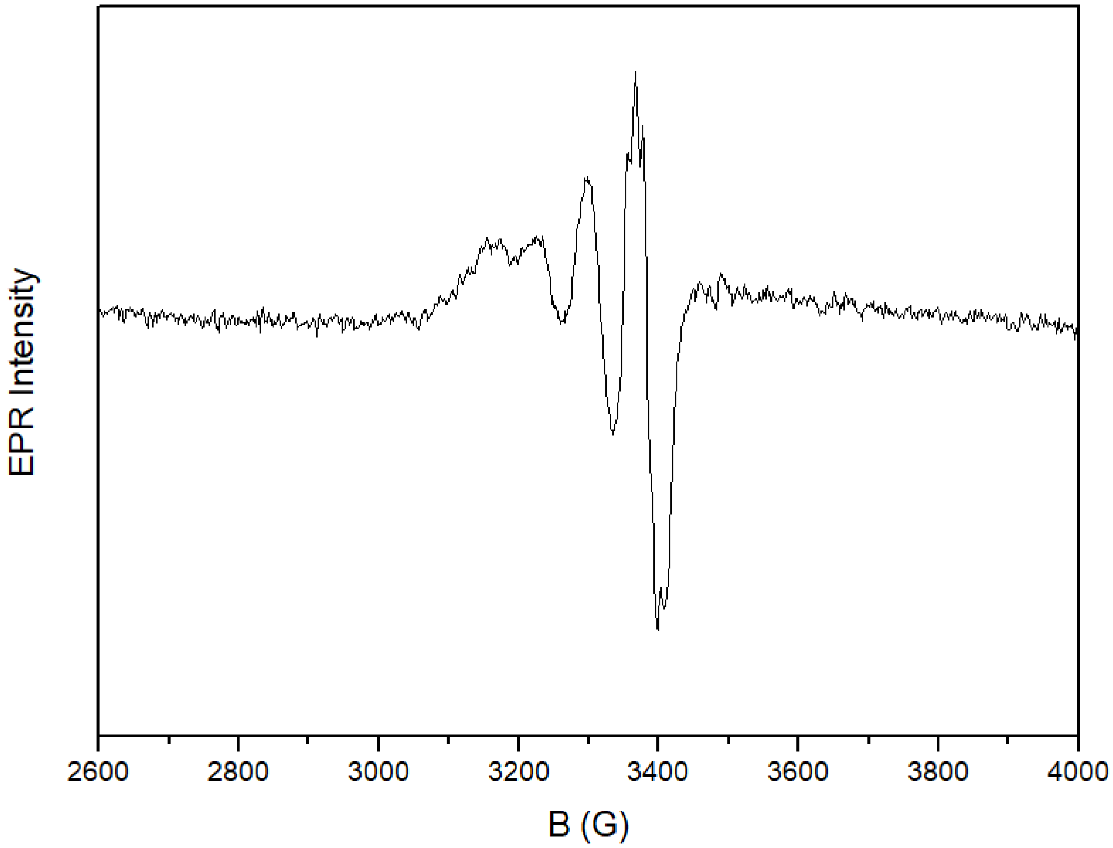

Figure S34. EPR spectrum of the complex Cu (**5**) at 10mM in methanol.

Part VIII- Stability in solution

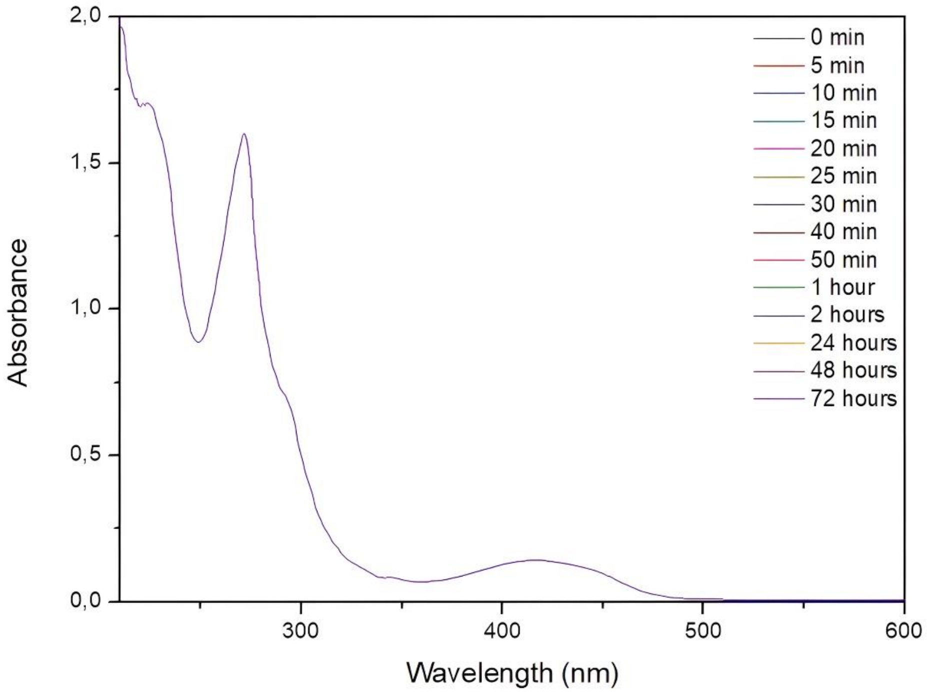

Figure S35. Uv-vis spectra as a function of time, of complex Cu (**1**) at 10^{-3} M in tris-HCl buffer (pH 7.4) containing 1% DMSO.

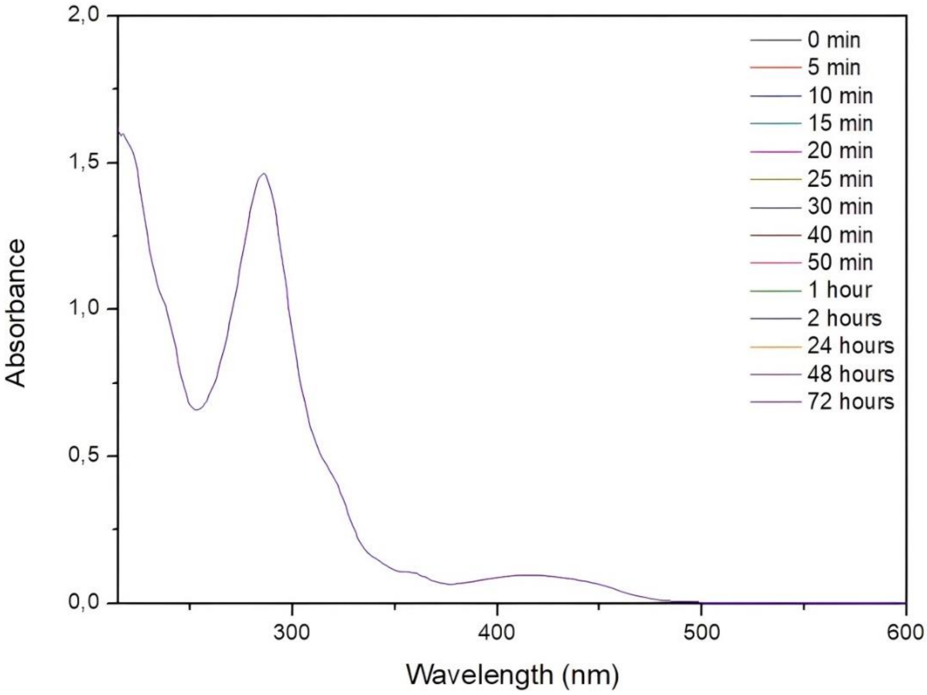

Figure S36. Uv-vis spectra as a function of time, of complex Cu (**2**) at 10^{-3} M in tris-HCl buffer (pH 7.4) containing 1% DMSO.

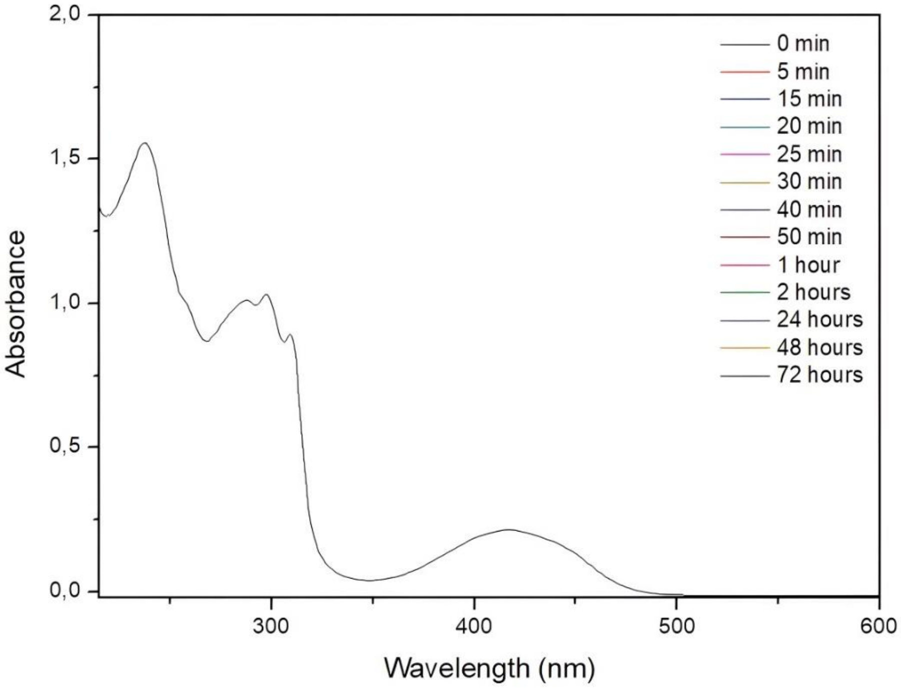

Figure S37. Uv-vis spectra as a function of time, of complex Cu (**3**) at 10^{-3} M at 10^{-3} M in tris-HCl buffer (pH 7.4) containing 1% DMSO.

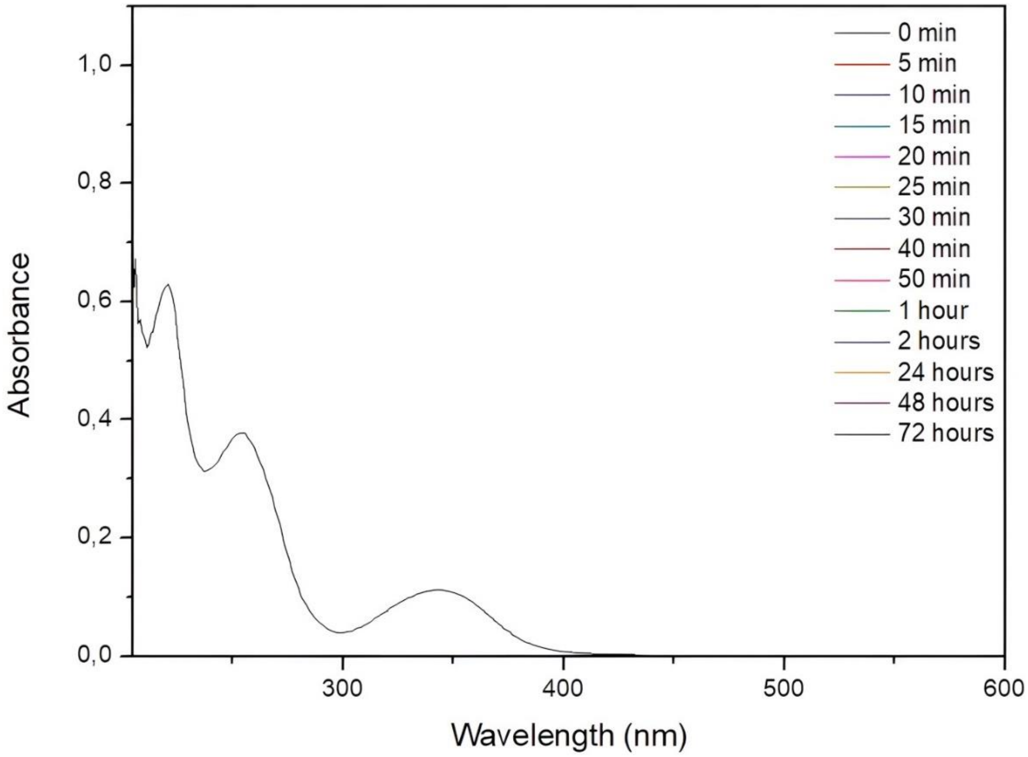

Figure S38. Uv-vis spectra as a function of time, of complex Cu (**4**) at at 10^{-3} M in tris-HCl buffer (pH 7.4) containing 1% DMSO.

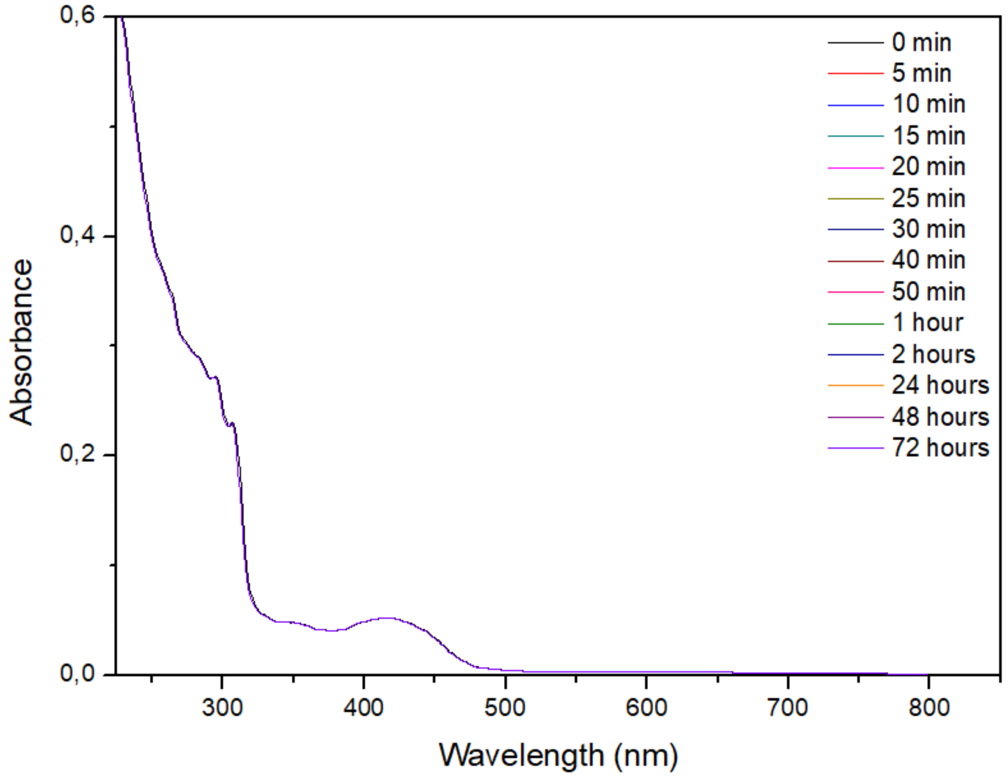

Figure S39. Uv-vis spectra as a function of time, of complex Cu (**5**) at at 10^{-3} M in tris-HCl buffer (pH 7.4) containing 1% DMSO.

Part IX – Agarose Gel Electrophoresis

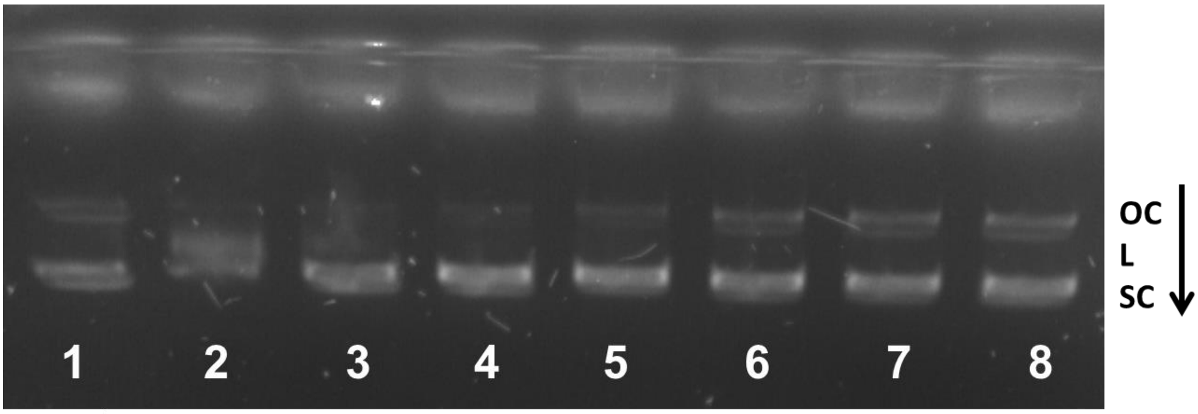

Figure S40. Effect of Cu (**2**) on pBR322 plasmid DNA mobility. Agarose gel electrophoresis of pBR322 (100 µM, in 10% DMSO) incubated for 2 h with increasing concentrations of the metal complex. Lane 1: DNA. Lane 2: cisplatin (100 µM). Lanes: 3–8: DNA + Cu (**2**) (10, 20, 40, 60, 80 and 100 µM).
